# Oxygenation thresholds for invasive ventilation in hypoxemic respiratory failure: a target trial emulation in two cohorts

**DOI:** 10.1186/s13054-023-04307-x

**Published:** 2023-02-22

**Authors:** Christopher J. Yarnell, Federico Angriman, Bruno L. Ferreyro, Kuan Liu, Harm Jan De Grooth, Lisa Burry, Laveena Munshi, Sangeeta Mehta, Leo Celi, Paul Elbers, Patrick Thoral, Laurent Brochard, Hannah Wunsch, Robert A. Fowler, Lillian Sung, George Tomlinson

**Affiliations:** 1 Keenan Research Centre for Biomedical Research, Li Ka Shing Knowledge Institute, St Michael’s Hospital, Unity Health Toronto, Toronto, Canada; 2 Interdepartmental Division of Critical Care Medicine, University of Toronto, Toronto, Canada; 3 Department of Medicine, Division of Respirology, University Health Network and Sinai Health System, Toronto, Canada; 4 Department of Pharmacy and Medicine, Sinai Health System, Toronto, Canada; 5 Department of Medicine, University of Toronto, Toronto, Canada; 6 Department of Medicine, University Health Network and Sinai Health System, Toronto, Canada; 7 Institute for Clinical Evaluative Sciences, Toronto, Canada; 8 Institute of Health Policy, Management and Evaluation, University of Toronto, Medical-Surgical ICU, 10th floor, 585 University Avenue, Toronto, ON M5G 1X5 Canada; 9 Leslie Dan Faculty of Pharmacy and Interdepartmental Division of Critical Care, University of Toronto, Toronto, ON Canada; 10 Sunnybrook Health Sciences Centre, Toronto, Canada; 11 Institute for Medical Engineering and Science, Massachusetts Institute of Technology, Cambridge, MA 02142 USA; 12 Division of Pulmonary, Critical Care and Sleep Medicine, Beth Israel Deaconess Medical Center, Boston, MA 02215 USA; 13 Department of Biostatistics, Harvard T.H. Chan School of Public Health, Boston, MA 02115 USA; 14 Department of Intensive Care Medicine, Laboratory for Critical Care Computational Intelligence, Amsterdam Medical Data Science, Amsterdam UMC, Vrije Universiteit, Amsterdam, The Netherlands; 15 Division of Haematology/Oncology, The Hospital for Sick Children, Toronto, Canada

**Keywords:** Hypoxemic respiratory failure, Intensive care medicine, Mechanical ventilation, Noninvasive ventilation, Thresholds for invasive ventilation, Target trial emulation, Bayesian analysis, Statistical methods

## Abstract

**Background:**

The optimal thresholds for the initiation of invasive ventilation in patients with hypoxemic respiratory failure are unknown. Using the saturation-to-inspired oxygen ratio (SF), we compared lower versus higher hypoxemia severity thresholds for initiating invasive ventilation.

**Methods:**

This target trial emulation included patients from the Medical Information Mart for Intensive Care (MIMIC-IV, 2008–2019) and the Amsterdam University Medical Centers (AmsterdamUMCdb, 2003–2016) databases admitted to intensive care and receiving inspired oxygen fraction ≥ 0.4 via non-rebreather mask, noninvasive ventilation, or high-flow nasal cannula. We compared the effect of using invasive ventilation initiation thresholds of SF < 110, < 98, and < 88 on 28-day mortality. MIMIC-IV was used for the primary analysis and AmsterdamUMCdb for the secondary analysis. We obtained posterior means and 95% credible intervals (CrI) with nonparametric Bayesian G-computation.

**Results:**

We studied 3,357 patients in the primary analysis. For invasive ventilation initiation thresholds SF < 110, SF < 98, and SF < 88, the predicted 28-day probabilities of invasive ventilation were 72%, 47%, and 19%. Predicted 28-day mortality was lowest with threshold SF < 110 (22.2%, CrI 19.2 to 25.0), compared to SF < 98 (absolute risk increase 1.6%, CrI 0.6 to 2.6) or SF < 88 (absolute risk increase 3.5%, CrI 1.4 to 5.4). In the secondary analysis (1,279 patients), the predicted 28-day probability of invasive ventilation was 50% for initiation threshold SF < 110, 28% for SF < 98, and 19% for SF < 88. In contrast with the primary analysis, predicted mortality was highest with threshold SF < 110 (14.6%, CrI 7.7 to 22.3), compared to SF < 98 (absolute risk decrease 0.5%, CrI 0.0 to 0.9) or SF < 88 (absolute risk decrease 1.9%, CrI 0.9 to 2.8).

**Conclusion:**

Initiating invasive ventilation at lower hypoxemia severity will increase the rate of invasive ventilation, but this can either increase or decrease the expected mortality, with the direction of effect likely depending on baseline mortality risk and clinical context.

**Supplementary Information:**

The online version contains supplementary material available at 10.1186/s13054-023-04307-x.

## Background

Acute hypoxemic respiratory failure affects 20–50% of patients admitted to an intensive care unit (ICU) [1–3]. Affected patients have a 20–40% mortality risk and survivors may experience decreased quality of life [1, 4–6]. Invasive ventilation is a potentially lifesaving intervention that restores compensated gas exchange [7, 8]. However, invasive ventilation exposes patients to the risks of peri-intubation cardiac arrest, ventilator-induced lung injury, pneumonia, delirium, and ICU-acquired weakness [9–13]. The best physiologic thresholds for the initiation of invasive ventilation are unknown [8].

Current practice varies. In qualitative research, clinicians factor multiple variables such as the degree of hypoxemia, work of breathing, and experience of the team into their decision for invasive ventilation [14, 15]. Randomized trials use multiple criteria incorporating hemodynamics, neurologic function, and respiratory status [16]. Observational cohorts show a low incidence of invasive ventilation after meeting various physiologic thresholds, including those used in trials [17], and profound inter-hospital variation in the use of invasive ventilation [18, 19]. Some of the observed practice patterns may cause harm through either overuse or delay in the initiation of invasive ventilation.

Relevant potential thresholds include the degree of hypoxemia, the criteria used in randomized trials, and thresholds that incorporate work of breathing, duration of respiratory failure, or clinical trajectory [20–23]. A randomized controlled trial would be the most robust design to compare outcomes according to threshold, but this trial is not feasible at present due to disagreement on the region of equipoise and uncertainty about which thresholds to test [24]. A target trial emulation is an observational study design for causal inference which strives to mirror the eligibility criteria and interventions of the corresponding randomized trial, when that trial cannot be easily performed [25, 26]. Using the saturation-to-inspired oxygen ratio (SF), we performed a target trial emulation to compare the effect of using invasive ventilation initiation thresholds of SF < 110, < 98, and < 88 on 28-day mortality.

## Methods

### Study design, setting, and oversight

This retrospective cohort study was structured as a target trial emulation (Additional file 1: Table e1). The study incorporated two deidentified patient-level databases of intensive care unit admissions: Medical Information Mart for Intensive Care IV (MIMIC-IV) [27, 29] and the Amsterdam University Medical Centers database (AmsterdamUMCdb) [30, 31]. MIMIC-IV includes 76,540 ICU admissions from Beth Israel Deaconess Medical Centre (BIDMC) in Boston, USA (2008–2019), and AmsterdamUMCdb includes 23,106 ICU admissions from Amsterdam University Medical Centers (Amsterdam UMC) in Amsterdam, The Netherlands (2003–2016). MIMIC-IV included more patients and a more comprehensive set of potential confounders, so it was used for the primary analysis while AmsterdamUMCdb was used for the secondary analysis. The University of Toronto research ethics board approved the protocol (#42,081). The Strengthening the Reporting of Observational Studies in Epidemiology (STROBE) checklist is in the Additional file 1: (§1) [32].

### Cohort

Patients became eligible when they were first documented to be receiving oxygen with inspired oxygen fraction (FiO2) of 0.4 or more via non-rebreather mask, noninvasive positive pressure ventilation (NIV), or high-flow nasal cannula (HFNC), within 24 h of ICU admission. We excluded patients with prior invasive ventilation during the same ICU admission, goals of care precluding invasive ventilation, ICU admission from the operating room, or a tracheostomy. Patients were also excluded when equipoise was less certain at the moment of eligibility, defined as a Glasgow Coma Scale (GCS) motor component of less than 4, or a partial pressure of carbon dioxide (pCO2) of 60 or more with pH of 7.20 or less [33]. Patients were not excluded if these characteristics developed during the follow-up period, after initial inclusion. Wherever oxygen flow was available but FiO2 was not (for example, non-rebreather masks), FiO2 was estimated using the validated equation: FiO2 = 0.21 + (oxygen flow in liters per minute)*0.03 [34]. Further details are available in Additional file 1: (§4, Table e2).

### Variables

Baseline variables were demographics (age, sex, race/ethnicity), ICU admission information (type of ICU, year of ICU admission), comorbidities, and baseline laboratory, clinical, and procedural data (Additional file 1: Figure e1) Time-varying covariates included heart rate, systolic blood pressure, vasopressor use, respiratory rate, peripheral oxygen saturation, inspired oxygen fraction (FiO2), oxygen device, GCS, abnormal work of breathing, pH, lactate, and partial pressure of carbon dioxide. HFNC was not used at Amsterdam UMC during the years of available data. AmsterdamUMCdb also lacked patient race/ethnicity, comorbidities, and work of breathing. Cohort extraction used Google BigQuery and R (https://doi.org/10.5281/zenodo.7314132).(35).

### Thresholds

The main analysis compared three thresholds for initiation of invasive ventilation: saturation-to-inspired oxygen ratio (SF) of < 110, < 98, and < 88. We chose the SF ratio because it is a simple yet accurate measure of hypoxemia that is applicable to acute care settings worldwide, and can be measured without causing pain or discomfort to patients [36, 37]. Lower SF values indicate more severe hypoxemia. The three target values correspond to steeper parts of the oxyhemoglobin dissociation curve at high inspired oxygen fractions: SF < 88 reflects a patient unable to maintain oxygen saturation 88% on FiO2 1.0; SF < 98 reflects a patient unable to maintain saturation 88% on FiO2 0.9 or saturation 98% on FiO2 1.0, and SF < 110 reflects a patient unable to maintain saturation 88% on FiO2 0.8 or saturation 98% on FiO2 0.9.

As an exploratory analysis, we included six physiologic thresholds from four other measures of hypoxemic respiratory failure: respiratory rate, work of breathing, hypoxemia duration, and hypoxemia trajectory (Additional file 1). Based on the thresholds used in randomized trials, we included two thresholds requiring multi-organ involvement. We also included a usual care threshold, where treatment was assigned using the time-varying probability of invasive ventilation from the confounder model (Additional file 1: §8.1).

We reported invasive ventilation use for all thresholds. Invasive ventilation occurred either after meeting a threshold during the 96-h target trial period, or in the course of usual care following the 96-h period. This meant that all thresholds were evaluated on all patients, and we anticipated higher rates of invasive ventilation for lower severity thresholds (such as SF < 110) because whenever a patient attained SF < 88 or SF < 98 (higher degrees of hypoxemia severity), they also had SF < 110.

**Fig. 1:**
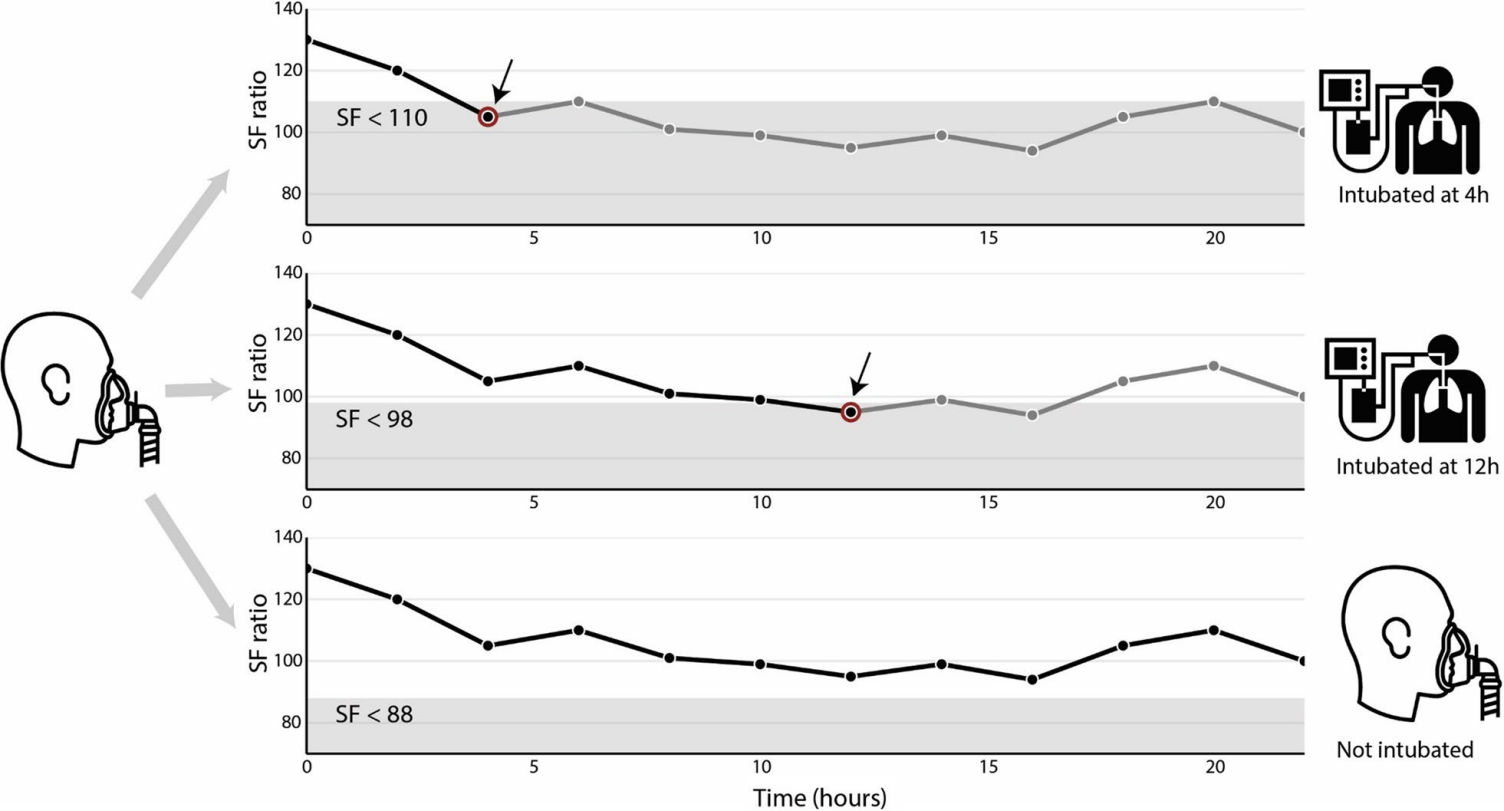
Oxygenation thresholds for initiating invasive ventilation. This figure explains how the saturation-to-inspired oxygen ratio (SF) threshold work. We begin with a patient on non-invasive oxygen support (left). Every threshold is tested on this patient (three gray arrows). The patient has the same underlying progression of SF ratios, independent of the choice of threshold (each line graph of SF versus time is identical). Top (SF < 110): At the first observation of SF < 110 (hour 4, red), the patient is intubated. Subsequent SF are not observed (grey), Middle (SF < 98 ): SF ≥98 until hour 12 (red), at which point the patient is intubated. Subsequent SF are not observed (grey). Bottom (SF < 88): SF ratio remains 88 or greater, so the patient remains on non-invasive oxygen support. All SF are observed

Note that the choice of threshold does not impact each patient’s SF ratios. Instead, each patient is modeled to either (1) receive invasive ventilation at the moment at which their SF ratio drops below the threshold under evaluation or (2) not receive invasive ventilation during the target trial period, if they get to the end of the 96-h target trial period without having an SF ratio below the threshold in question (Fig. 1).

### Observation schedule and follow-up

The thresholds were active until the earliest of invasive ventilation, ICU discharge, death, or 96 h from eligibility. During the 96-h target trial period, patients were evaluated every 2 hours for meeting thresholds for invasive ventilation. Patients were followed until the earliest of death, hospital discharge, or 28 days from eligibility.

### Outcomes and subgroup analyses

The primary outcome was 28-day mortality. We incorporated subgroup analyses according to sex, race/ethnicity, age, admission year, weight, initial oxygen device, and initial inspired oxygen fraction.

### Statistical analysis

We used nonparametric Bayesian G-computation to predict the causal effect of each threshold on outcomes [38–40]. G-computation is an established method to calculate unbiased treatment effects in observational study designs with time-varying confounding [41]. It uses two-component models, known as the confounder model and the conditional outcome model. Like all models for causal inference from observational data, the validity of the results depends on meeting the assumptions of positivity (every patient could potentially receive invasive ventilation), no interference (one patient’s use of invasive ventilation does not affect another patient’s use), consistency (well-defined intervention), and no unmeasured confounding [41].

The confounder model estimated the relationship between previously observed and future values of time-varying confounding variables, for patients not invasively ventilated. For this model, we used a Hilbert space Gaussian process approximation (Additional file 1: §6) [42–45]. A Gaussian process is a nonparametric Bayesian model that allows covariance across time (the past can influence the future) and between variables (different variables, such as heart rate or respiratory rate, can influence each other). Modeling the data as a Gaussian process amounts to assuming that there is an underlying smooth process (disease trajectory) that is observed over time through each of the continuous and discrete clinical variables. This approach is desirable because it can model complex confounding relationships and account for the associations between covariates. We assessed the validity of the confounder model through the measurement of prediction error (continuous variables) and discrimination/precision (binary variables) on data not used to fit the model.

The conditional outcome model estimated the probability of 28-day mortality, conditional on observing a sequence of confounder variables and invasive ventilation status. We used Bayesian additive regression trees (BART), a nonparametric model that sums results from multiple classification trees. Prior distributions encourage small, simple trees with regularized leaf weights. BART can effectively describe nonlinear relationships and interactions between outcomes and confounders and has demonstrated success compared to other models in estimating confounded treatment effects [46–50]. We calculated the model’s discrimination, precision, and calibration using fivefold cross-validation.

The nonparametric G-formula combined the two models and treatment thresholds to generate predictions of the effects of the thresholds on mortality (Additional file 1). While in a true randomized trial, each participant is randomized to only one treatment, in this target trial emulation we predict outcomes for every threshold on every patient. For each threshold, we reported the probability of each outcome and an odds ratio for mortality in comparison with modeled usual care, all summarized by their means and 95% credible intervals (CrI). We calculated e-values to quantify the strength of unmeasured confounding required to negate the findings [51, 52]. We used 400 samples from the posterior distribution. Programming was done in R v4.0.3 [35] and Stan [53] using the Niagara computer cluster from the Digital Research Alliance Canada [54]. All code is available at https://doi.org/10.5281/zenodo.7314132.

## Results

The primary analysis included 3,357 patients from MIMIC-IV (Additional file 1: Figure e9). The median age was 65 (interquartile range (IQR) 58 to 79) years and 45% (1,500) were women (Table 1). Most (63%) were admitted to a medical or surgical ICU. At eligibility, 16% (536) were using HFNC, 14% (483) NIV, and 70% (2,338) non-rebreather masks. The median baseline SF was 148 (IQR 136 to 174). Within 28 days, 896 patients (26.7%) received invasive ventilation and 745 patients (22.2%) died. Mortality was 17.7% in patients who did not receive invasive ventilation and 34.5% in patients who received invasive ventilation.

**Table 1:** Primary analysis cohort (MIMIC-IV) characteristics

	Total (*N* (%))	Oxygen device in use at eligibility (*N* (%))		
		High-flow nasal cannula	Noninvasive ventilation	Non-rebreather mask
Total	3,357	536 (16)	483 (14)	2,338 (70)
*Age-group (years)*				
18–39	220 (7)	39 (7)	27 (6)	154 (7)
40–49	259 (8)	41 (8)	32 (7)	186 (8)
50–59	575 (17)	100 (19)	89 (18)	386 (17)
60–69	745 (22)	128 (24)	121 (25)	496 (21)
70–79	742 (22)	127 (24)	120 (25)	495 (21)
80 or more	816 (24)	101 (19)	94 (20)	621 (27)
*Sex*				
Female	1,500 (45)	223 (42)	232 (48)	1,045 (45)
Male	1,857 (55)	313 (58)	251 (52)	1,293 (55)
*Race/ethnicity*				
White	2,396 (71)	395 (74)	326 (68)	1,675 (72)
Black	342 (10)	29 (5)	71 (15)	242 (10)
Hispanic	117 (4)	17 (3)	19 (4)	81 (4)
Asian	93 (3)	14 (3)	5 (1)	74 (3)
Other / unknown	409 (12)	81 (15)	62 (13)	266 (11)
*Year of ICU admission*				
2008 – 2010	1,409 (42)	106 (20)	166 (34)	1,137 (49)
2011 – 2013	740 (22)	66 (12)	90 (19)	584 (25)
2014 – 2016	635 (19)	126 (24)	113 (23)	396 (17)
2017 – 2019	573 (17)	238 (44)	114 (24)	221 (10)
*Intensive care unit type*				
Cardiac	807 (24)	85 (16)	135 (28)	587 (25)
Medical-surgical	2,117 (63)	396 (74)	304 (63)	1,417 (61)
Neuro-trauma	433 (13)	55 (10)	44 (9)	334 (14)
*Comorbidities*				
Chronic obstructive pulmonary disease	696 (21)	148 (28)	175 (36)	373 (16)
Congestive heart failure	1,435 (43)	198 (37)	285 (59)	952 (41)
*Baseline clinical variables (median [IQR])*				
Peripheral oxygen saturation	96 [93, 99]	94 [92, 97]	95 [92, 98]	96 [93, 99]
Fraction of inspired oxygen	0.66 [0.57, 0.68]	0.70 [0.60, 1.0]	0.51 [0.48, 0.66]	0.66 [0.57, 0.66]
Saturation-to-inspired oxygen (SF) ratio	148 [136, 174]	131 [98, 165]	186 [145, 200]	147 [139, 164]
Respiratory rate	23 [19, 28]	23 [20, 28]	23 [19, 29]	23 [19, 27]
*Oxygen device used at any point*				
High-flow nasal cannula	818 (24)	536 (100)	75 (16)	207 (9)
Noninvasive ventilation	729 (22)	57 (11)	483 (100)	189 (8)
Non-rebreather	2,741 (82)	210 (39)	195 (40)	2,338 (100)

This table shows the baseline characteristics for the cohort used in the primary analysis (MIMIC-IV cohort), grouped by oxygen device in use at time of initial eligibility.

*ICU* Intensive care unit, *IQR* Interquartile range

### Predicted probabilities of invasive ventilation by threshold

The predicted probabilities of invasive ventilation and mortality at 28 days were calculated using G-computation for all thresholds in all patients, and model diagnostics and cross-validation are available in Additional file 1. The mean predicted probability of invasive ventilation at 28 days was 71.8% with a threshold of SF < 110, 47.0% with a threshold of SF < 98, and 19.4% with a threshold of SF < 88.

### Mortality by threshold

**Fig. 2:**
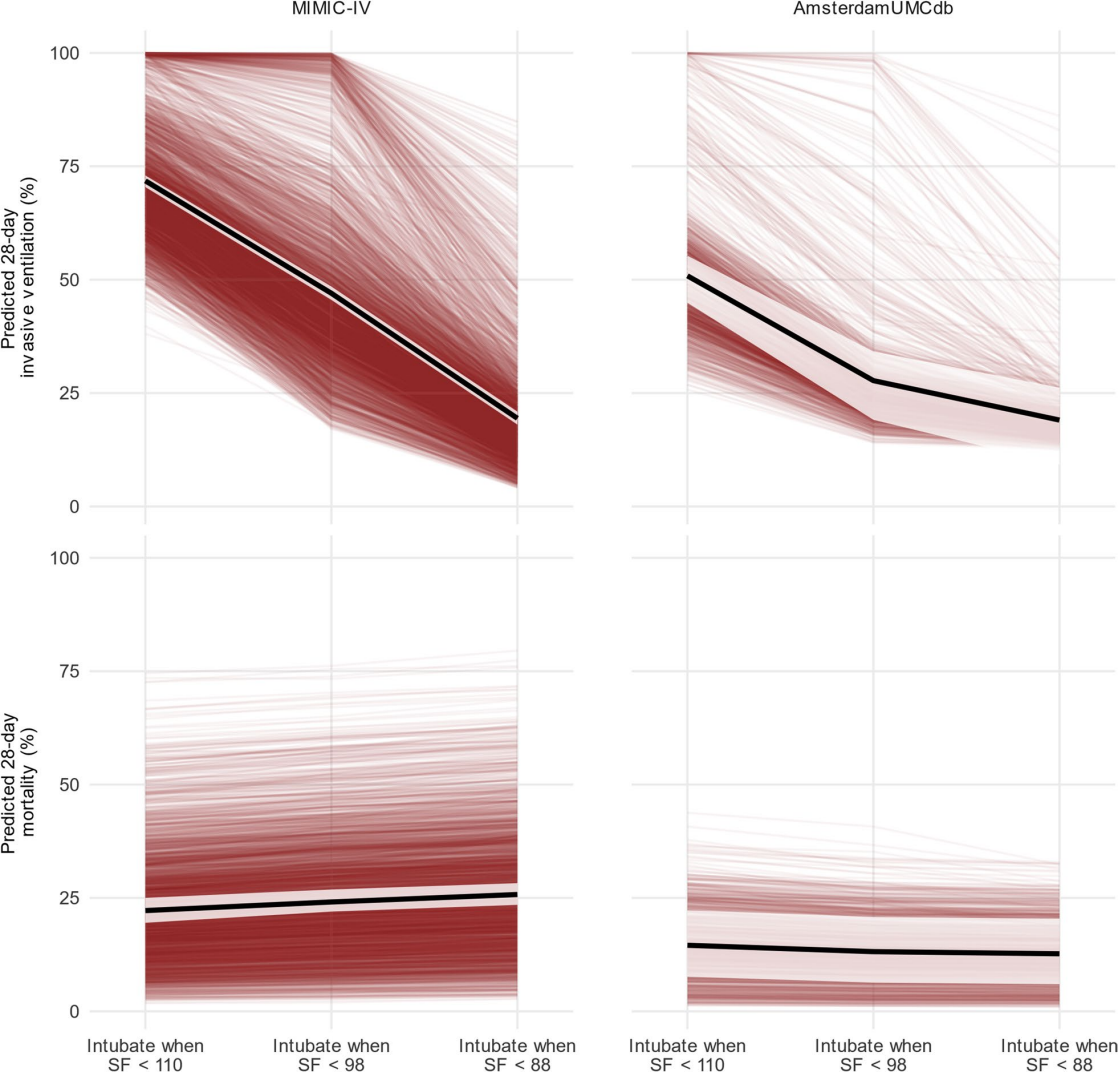
Predicted 28-day probabilities of invasive ventilation and mortality by SF ratio threshold for initiating invasive ventilation. This figure shows the predicted 28-day probability (y-axis) of invasive ventilation (top) and mortality (bottom), for the primary (MIMIC-IV, left) and secondary (AmsterdamUMCdb, right) analyses, according to each threshold trigger for invasive ventilation (x-axis). The mean predicted probability is in black, 95% credible interval in white, and red lines show the mean predicted probability for each of the 3,357 (MIMIC-IV) or 1,279 (AmsterdamUMCdb) individual patients, allowing for inspection of results across thresholds for each patient. The predicted probability of invasive ventilation increases dramatically with higher SF ratio thresholds for invasive ventilation, while the predicted probability of mortality decreases slightly for MIMIC-IV and increases slightly for AmsterdamUMCdb. The variation between patients is greater than the variation between thresholds, especially for mortality

The mean predicted 28-day mortality according to invasive ventilation threshold was 22.2% with a threshold of SF < 110, 24.1% with a threshold of SF < 98, and 25.8% with a threshold of SF < 88 (Fig. 2). Compared to a threshold of SF < 110, the absolute risk increases were 1.6% (CrI 0.6 to 2.6) with a threshold of SF < 98 and 3.5% (CrI 1.4 to 5.4) with a threshold of SF < 88. Using a threshold of SF < 110 instead of a threshold of SF < 88 was associated with 1 additional survivor for every 15 (CrI 10 to 39) additional patients invasively ventilated.

### Comparison with usual care

**Fig. 3:**
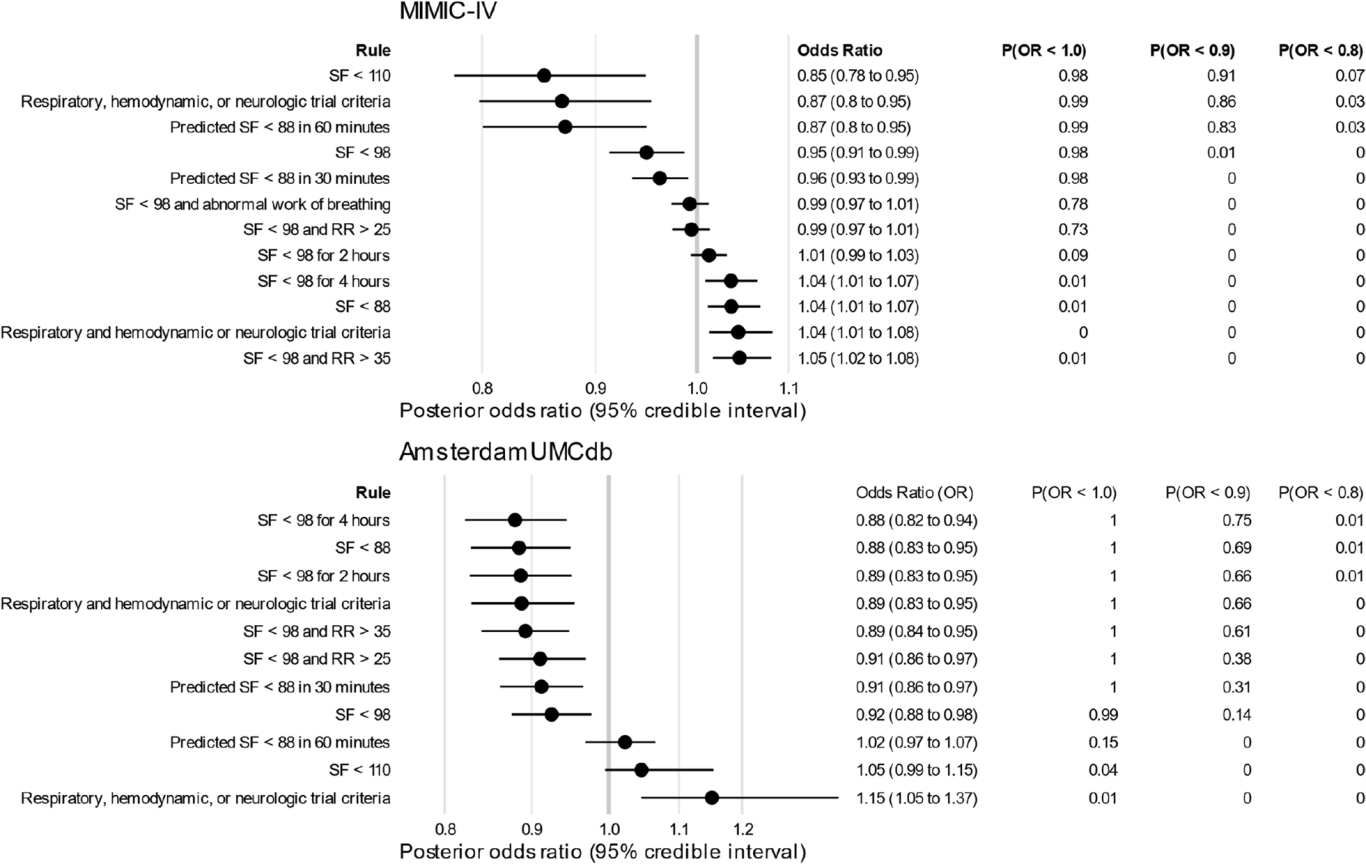
Odds ratios for mortality of each threshold in comparison with usual care. For both primary analysis (MIMIC-IV) and secondary analysis (AmsterdamUMCdb), this figure shows the posterior odds ratios (mean and 95% credible interval) for 28-day mortality alongside the probabilities that the posterior odds ratio (OR) is less than 1 (P(OR < 1.0)), less than 0.9 (P(OR < 0.9)), and less than 0.8 (P(OR < 0.8)). The reference threshold is usual care (OR = 1). SF = saturation-to-inspired oxygen fraction ratio, RR = respiratory rate. Respiratory trial criteria were 2 of RR > 40, saturation < 90 on inspired oxygen 0.90 or higher, abnormal work of breathing, or pH < 7.35; hemodynamic criterion was use of vasopressors; neurologic criterion was Glasgow Coma Scale < 9. Predicted SF was calculated using linear extrapolation between the current and previous SF measurements (measurements occurred every 2 h)

The threshold based on usual care resulted in predicted 28-day probabilities of 31.5% for invasive ventilation and 25.1% for mortality. Compared to usual care, the odds ratio for 28-day mortality was 0.85 (0.78 to 0.95) with a threshold of SF < 110, 0.95 (CrI 0.91 to 0.99) with a threshold of SF < 98, and 1.04 (CrI 1.01 to 1.08) with a threshold of SF < 88 (Fig. 3). For all thresholds, it was very unlikely (probability 7% or less) that the odds ratio for mortality was less than 0.8 (Table 2).

**Table 2:** Secondary analysis cohort (AmsterdamUMCdb) characteristics

	Total (*N* (%))	Oxygen device in use at eligibility (*N* (%))	
	All	Noninvasive ventilation	Non-rebreather mask
Total	1,279	296	983
*Age-group*			
18–39	131 (10)	19 (6)	112 (11)
40–49	103 (8)	15 (5)	88 (9)
50–59	213 (17)	46 (16)	167 (17)
60–69	312 (24)	84 (28)	228 (23)
70–79	342 (27)	89 (30)	253 (26)
80 +	178 (14)	43 (15)	135 (14)
*Sex*			
Female	493 (39)	123 (42)	370 (38)
Male	786 (61)	173 (58)	613 (62)
*Year of ICU admission*			
2003–2009	801 (63)	204 (69)	597 (61)
2010–2016	478 (37)	92 (31)	386 (39)
*Type of care unit*			
Intensive care (Level 3)	908 (71)	236 (80)	672 (68)
Medium care (Level 2)	145 (11)	42 (14)	103 (11)
Both	210 (16)	17 (6)	193 (20)
*Baseline clinical data (median [IQR])*			
Peripheral oxygen saturation	96 [93, 98]	95 [92, 98]	96 [93, 98]
Fraction of inspired oxygen	0.60 [0.51, 0.66]	0.60 [0.42, 0.77]	0.66 [0.51, 0.66]
Respiratory rate	28 [22, 34]	31 [25, 38]	25 [20, 32]
*Oxygen device used at any point*			
Noninvasive ventilation	472 (37)	296 (100)	176 (18)
Non-rebreather mask	1,119 (88)	139 (47)	983 (100)

This table shows the baseline characteristics for the cohort used in the secondary analysis (AmsterdamUMCdb cohort), grouped by oxygen device in use at time of initial eligibility.

*ICU* Intensive care unit, *IQR* Interquartile range

### Additional thresholds

Across four additional dimensions of hypoxemic respiratory failure (respiratory rate, work of breathing, duration, trajectory), thresholds triggering invasive ventilation at a lower severity resulted in more predicted invasive ventilation and less predicted mortality (Table 3). The randomized trial criteria threshold requiring any respiratory, hemodynamic, or neurologic dysfunction resulted in predicted 28-day probabilities of 65.5% for invasive ventilation and 22.5% for mortality, while a threshold of respiratory dysfunction in combination with either hemodynamic or neurologic dysfunction resulted in predicted 28-day probabilities of 16.1% for invasive ventilation and 25.9% for mortality.

**Table 3:** Mean predicted 28-day probabilities of invasive ventilation and mortality

	Invasive ventilation (28-day)		Mortality (28-day)	
Threshold	MIMIC-IV	AmsterdamUMCdb	MIMIC-IV	AmsterdamUMCdb
*Oxygenation thresholds*				
SF < 110	71.8 (70.4 to 73.0)	50.9 (44.9 to 55.3)	22.2 (19.5 to 25.0)	14.6 (7.7 to 22.3)
SF < 98	47.0 (45.6 to 48.5)	27.7 (19.1 to 34.2)	24.1 (22.0 to 26.9)	13.2 (6.3 to 20.8)
SF < 88	19.4 (18.0 to 20.9)	19.0 (9.2 to 26.2)	25.8 (23.5 to 28.3)	12.7 (6.0 to 20.4)
*Usual care*				
Observed	26.7 (25.2 to 28.2)	36.8 (34.1 to 39.5)	22.2 (20.8 to 23.6)	17.4 (15.3 to 19.6)
Modeled	31.5 (29.7 to 33.5)	45.7 (38.4 to 50.7)	25.1 (22.9 to 27.8)	14.1 (6.7 to 22.3)
*Secondary outcomes: respiratory rate*				
SF < 98 and RR > 25	32.9 (31.4 to 34.4)	24.6 (15.4 to 31.4)	25.0 (22.9 to 27.7)	13.0 (6.3 to 20.7)
SF < 98 and RR > 35	16.3 (15.0 to 17.6)	21.0 (11.3 to 27.9)	25.9 (23.7 to 28.5)	12.8 (6.1 to 20.8)
*Secondary outcomes: work of breathing*				
SF < 98 and abnormal work of breathing	33.5 (31.9 to 35.2)	–	24.9 (22.8 to 27.7)	–
*Secondary outcomes: duration*				
SF < 98 for 2 consecutive hours	27.6 (26.0 to 29.0)	19.9 (10.2 to 26.9)	25.3 (23.2 to 28.1)	12.7 (5.9 to 20.7)
SF < 98 for 4 consecutive hours	19.8 (18.4 to 21.2)	18.1 (8.1 to 25.3)	25.8 (23.6 to 28.3)	12.6 (5.9 to 20.4)
*Secondary outcomes: trajectory*				
SF predicted to be less than 88 in 30 min	67.5 (66.2 to 68.8)	51.0 (44.8 to 55.3)	22.6 (20.1 to 25.4)	14.4 (6.7 to 22.9)
SF predicted to be less than 88 in 60 min	43.0 (41.5 to 44.4)	26.2 (17.1 to 32.9)	24.4 (22.3 to 27.2)	13.0 (6.1 to 21.0)
*Secondary outcomes: randomized trial criteria*				
Respiratory, hemodynamic, or neurologic trial criteria	65.5 (64.2 to 66.8)	64.7 (59.8 to 68.3)	22.5 (19.9 to 25.4)	15.7 (9.0 to 22.3)
Respiratory and hemodynamic or neurologic trial criteria	16.1 (14.8 to 17.5)	19.2 (9.2 to 26.5)	25.9 (23.7 to 28.6)	12.7 (6.1 to 20.4)

This table shows the posterior mean and the 95% credible interval of the mean by threshold for predicted 28-day invasive ventilation and mortality for the primary (MIMIC-IV) and secondary (AmsterdamUMCdb) analyses. Note that all thresholds were evaluated in all patients. *SF*  Saturation-to-fraction of inspired oxygen ratio, *RR*  respiratory rate, MIMIC-IV = Medical Information Mart for Intensive Care Version IV, AmsterdamUMCdb = Amsterdam University Medical Center database.

^*^Predicted SF was calculated using linear extrapolation between the current and previous SF measurements (measurements occurred every 2 h).

^**^Randomized trial criteria: respiratory criteria = 2 of RR > 40, saturation < 90 on inspired oxygen 0.90 or higher, abnormal work of breathing, or pH < 7.35; hemodynamic criteria = use of breathing, or pH < 7.35; hemodynamic criteria = use of vasopressors; neurologic criteria = Glasgow Coma Scale < 9.

### Subgroup analyses

**Fig. 4:**
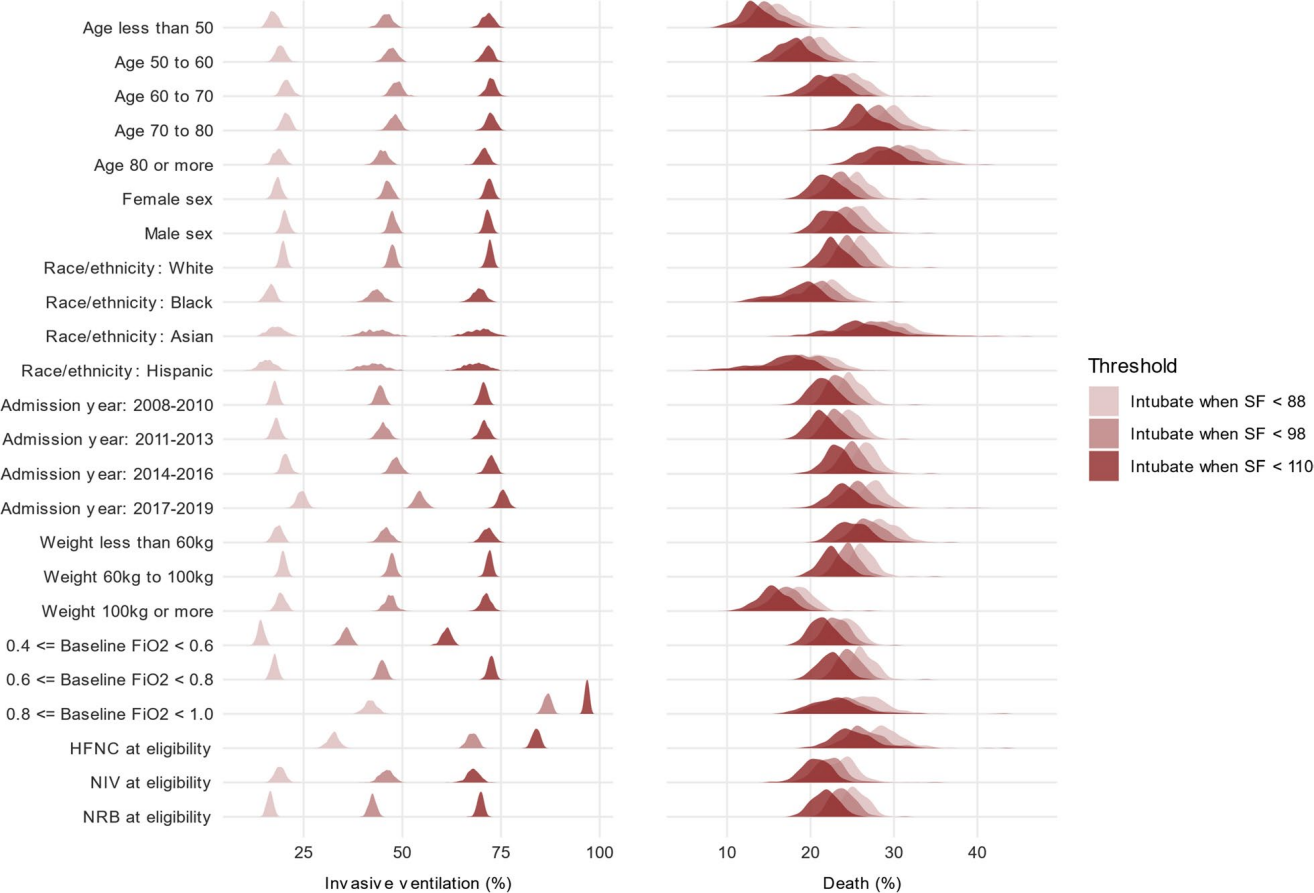
Mean predicted 28-day probabilities of invasive ventilation and mortality by subgroup. This figure shows the posterior probability densities of the mean predicted probabilities (x-axis) of invasive ventilation (left column) and mortality (right column) at 28 days according to threshold (light, medium, or dark densities) and subgroup (row) for the primary (MIMIC-IV) analysis. The ordering of results by threshold is consistent across subgroup for both invasive ventilation and mortality. The predicted probabilities of invasive ventilation by threshold are relatively stable across subgroups, except for baseline fraction of inspired oxygen or oxygen device at eligibility. The predicted probabilities of mortality vary according to baseline characteristics, including increases with increasing age or decreases with weight 100 kg or more

Results for the primary analysis showed consistency between the SF thresholds across the subgroups of age, sex, race/ethnicity, admission year, weight, baseline inspired oxygen fraction, and baseline oxygen device (Fig. 4). In all subgroups, predicted probability of invasive ventilation was lowest with threshold SF < 88, and predicted mortality was lowest with threshold SF < 110.

### Secondary analysis

The AmsterdamUMCdb cohort included 1,279 patients (Additional file 1: Figure e9); 39% (493) were women and the median age-group was 60–69 years (Table 3). Noninvasive ventilation was in use for 23% (296) at eligibility, while the remainder used non-rebreather masks. Within 28 days from eligibility, 470 patients (36.8%) received invasive ventilation and 222 patients (17.4%) died. Mortality was 14.6% in patients who did not receive invasive ventilation and 22.1% in patients who received invasive ventilation. Compared to the primary analysis, the secondary analysis incorporated fewer measured confounders and had worse discrimination, precision, and calibration (Additional file 1: Tables e3–e5, Figures e2–se8).

The mean predicted probability of 28-day invasive ventilation was 50.9% with a threshold of SF < 110, 27.7% with a threshold of SF < 98, and 19.0% with a threshold of SF < 110 (Table 2). The corresponding probabilities of 28-day mortality were 14.6%, 13.2%, and 12.7% (Fig. 2). Compared to a threshold of SF < 110, the absolute risk decrease was 0.5% (CrI 0.0 to 0.9) with a threshold of SF < 98 and 1.9% (CrI 0.9 to 2.8) with a threshold of SF < 88.

Modeled usual care resulted in a predicted 28-day invasive ventilation probability of 45.7% and a predicted 28-day mortality probability of 14.1%. The odds ratios of 28-day mortality for each threshold relative to usual care were 1.05 (CrI 0.99 to 1.15) with a threshold of SF < 110, 0.92 (CrI 0.88 to 0.98) with a threshold of SF < 98, and 0.88 (CrI 0.82 to 0.94) with a threshold of SF < 88 (Fig. 3).

## Discussion

This target trial emulation of thresholds for initiating invasive ventilation in hypoxemic respiratory failure showed that using a threshold of SF < 110 as a trigger to initiate invasive ventilation resulted in more predicted 28-day invasive ventilation than thresholds of SF < 98 or SF < 88. In the primary analysis, the predicted 28-day mortality was lowest with a threshold of SF < 110. Across additional thresholds focused on respiratory rate, work of breathing, duration of hypoxemia, trajectory of hypoxemia, and multi-organ criteria from randomized trials, thresholds met at lower as opposed to higher severity led to lower predicted mortality. By contrast, in the secondary analysis, predicted mortality was highest with a threshold of SF < 110, and across the additional thresholds, those met at lower severity were associated with higher predicted mortality.

The different relationship between invasive ventilation thresholds and mortality comparing primary and secondary analyses might be explained by differences in internal validity or clinical context. Compared to the secondary analysis, the primary analysis had more patients, more measured confounding variables, better discrimination, better precision, and better calibration. The direction of residual bias was harder to predict because the target trial construction did not permit death before invasive ventilation during the 96-h target trial period, which favored higher severity thresholds, but the possible inclusion of patients with care limitations favored lower severity thresholds.

Differences in the clinical context of the two cohorts may also explain the findings. The two sites differed in terms of time period, healthcare system, ICU beds, patient population, oxygen device availability, and clinical practice. The secondary analysis encompasses an older observation period where contemporary approaches to ventilation, weaning, and sedation may not have been employed, potentially increasing the harms associated with invasive ventilation. In the secondary analysis, no patients used high-flow nasal cannula; however, this would be expected to bias results toward finding invasive ventilation more beneficial, the opposite direction from our findings. At BIDMC patients rely on private medical insurance and ICU beds comprise 11% of total hospital beds, while at Amsterdam UMC there is universal coverage for hospital care and ICU beds comprise only 4.4% of hospital beds [55, 56]. The impact of ICU bed availability on the results is harder to predict, but one potential impact is that the decision for invasive ventilation may be more commonly made prior to ICU admission in Amsterdam UMC as compared to BIDMC.

The results support the hypothesis that the benefit of invasive ventilation is related to underlying disease severity. Mortality was lower in the AmsterdamUMCdb cohort, implying a lower disease severity for non-intubated patients in that database. Other research also supports this hypothesis. In an observational study, ARDS patients with arterial-to-inspired oxygen ratio less than 150 mmHg managed using invasive as opposed to noninvasive ventilation had lower mortality [21]. In patients with COVID-19, higher baseline sequential organ failure assessment scores were associated with better outcomes when patients were managed with invasive ventilation as opposed to noninvasive oxygen strategies [57]. For patients with higher predicted mortality, invasive ventilation thresholds triggered at a lower severity of illness could confer benefits by avoiding catastrophic deteriorations, emergency intubations, or patient self-inflicted lung injury [58, 59]. By contrast, for patients with lower predicted mortality, the benefits of avoiding iatrogenic complications associated with intubation and invasive ventilation may predominate.

However, not all research accords with this conclusion. Two small randomized trials from 1998 and 2005 suggested benefits with a higher severity threshold for invasive ventilation among a population with severe hypoxemic respiratory failure [60, 61]. Options for noninvasive oxygen support, and the use of contemporary best practices for ventilation, weaning, and sedation may have been reduced in those studies, highlighting that the balance of benefit and harm associated with invasive ventilation will depend on the use of noninvasive oxygen strategies and best practices during invasive ventilation [11, 62–65].

This study has important limitations. Unmeasured confounding is present because the clinical decision for invasive ventilation incorporates information about the diagnosis, prognosis, and nuanced respiratory assessment that are not available in the data studied. Unavoidably, the methods involved many modeling decisions which may affect the results in unpredictable ways [66]. The study does not report functional outcomes, where the harms of excess invasive ventilation may be more evident [6, 67]. The choice of SF ratio for the primary thresholds is problematic for people with darker skin pigment in whom peripheral oximeters can overestimate arterial oxygen saturation; we recommend using arterial oxygen saturation when a discrepancy is possible [68]. The target trial duration of 96 h captures most but not all intubations for hypoxemic respiratory failure [20, 69]. Some of the data were gathered more than 10–15 years ago and may not reflect current clinical practice.

The study also has considerable strengths. The methods are novel, fully documented, and address many challenges in correlating treatment decisions with clinical outcomes from retrospective data, including immortal time bias, indication bias, and time-varying confounding [66, 67]. The predictive validity of the component models has been explicitly assessed and documented. The thresholds evaluated are simple and clinically applicable.

These results highlight many areas for future research. Optimal thresholds may additional physiologic data such as standardized dyspnea assessment, electrical impedance tomography, or esophageal manometry [22, 70, 71]. More complex thresholds could be found through reinforcement learning [72, 73]. More information is also needed to compare thresholds with respect to functional outcomes, cost-effectiveness, and patient preferences.

## Conclusion

For patients with hypoxemic respiratory failure, initiating invasive ventilation at lower hypoxemia severity will increase the rate of invasive ventilation, but this can either increase or decrease the expected mortality, with the direction of effect likely depending on baseline mortality risk and clinical context.

## Supplementary Information

**Additional file 1. **Supplementary material including further details about study design, data processing, statistical analysis, and results.

## Data Availability

The data used in this study were deidentified and are available through Physionet (MIMIC-IV) or the Amsterdam Medical Data Sciences organization (AmsterdamUMCdb). Both are freely available to researchers who complete a free basic online research ethics course and sign a data use agreement. See https://doi.org/10.13026/rrgf-xw32 for MIMIC-IV data access and https://amsterdammedicaldatascience.nl/amsterdamumcdb/ for AmsterdamUMCdb access.

## Abbreviations

ICU: Intensive care unit
MIMIC-IV: Medical information mart for intensive care version IV
AmsterdamUMCdb: Amsterdam University Medical Centers Database
BIDMC: Beth Israel Deaconess Medical Centre
Amsterdam UMC: Amsterdam University Medical Centers
STROBE: Strengthening The Reporting of Observational Studies in Epidemiology
NIV: Noninvasive positive pressure ventilation
HFNC: High-flow nasal cannula
GCS: Glasgow coma scale
pCO2: Partial pressure of carbon dioxide
FiO2: Inspired oxygen fraction
SF: Saturation-to-inspired oxygen ratio
BART: Bayesian additive regression trees
CrI: 95% Credible interval
