# Supplementary material for: Oxygenation thresholds for invasive ventilation in hypoxemic respiratory failure: a target trial emulation in two cohorts
Source: Crit Care. 2023 Feb 22;27:67. doi: 10.1186/s13054-023-04307-x (PMC9944781; doi:10.1186/s13054-023-04307-x)
Supplement: Supplementary file 1 — Additional file 1. Supplementary material including further details about study design, data processing, statistical analysis, and results. [file 13054_2023_4307_MOESM1_ESM.pdf]

Supplement – Oxygenation thresholds for invasive ventilation in hypoxemic respiratory failure: a target trial emulation in two cohorts

Yarnell, Christopher J MD; ORCID 0000-0001-5657-9398

Angriman, Federico; ORCID 0000-0003-0971-386X

Ferreyro, Bruno L MD; ORCID 000-0001-7485-3741

Liu, Kuan

De Grooth, Harm Jan

Burry, Lisa; ORCID 0000-0002-6545-3890

Munshi, Laveena

Mehta, Sangeeta ORCID 0000-0002-7073-4769

Celi, Leo

Elbers, Paul

Thoral, Patrick

Brochard, Laurent

Wunsch, Hannah

Fowler, Robert A MDCM

Sung, Lillian MD

Tomlinson, George PhD

2022-12-28

Contents

1 STROBE statement ... 4
2 Target trial details: Secondary thresholds ... 6
2.1.1 Respiratory rate ... 6
2.1.2 Work of breathing ... 6
2.1.3 Duration ... 6
2.1.4 Trajectory ... 6
2.1.5 Multi-organ involvement ... 7
2.1.6 Usual care ... 7
2.2 Table e1: Target trial characteristics ... 8
3 Cohort construction – MIMIC-IV ... 9
3.1 Eligibility assessment ... 9
3.2 Specific variables ... 9
3.2.1 Tracheostomies ... 9
3.2.2 Goals of care ... 9
3.2.3 Race/ethnicity ... 10
3.2.4 Care unit ... 10
3.2.5 Fraction of inspired oxygen ... 10
3.2.6 Work of breathing ... 11
4 Cohort construction – AmsterdamUMCdb ... 12
4.1 Eligibility assessment ... 12
4.2 Validated observations ... 12
4.3 Specific variables ... 12
4.3.1 Table e2: Oxygen devices ... 12
4.3.2 Goals of care ... 13
4.3.3 Operative ... 13
4.3.4 Invasive ventilation ... 13
4.3.5 Care unit ... 13
5 Using the G-formula in this study: rationale and explanation ... 14
6 Confounder model ... 14
6.1 Figure e1: Conceptual model ... 15
6.2 Multitask Gaussian processes in clinical medicine ... 15
6.3 Hilbert-space Gaussian process approximation ... 16
6.4 Data transformations ... 16
6.5 Model description ... 17

6.5.1 Mean function 17
6.5.2 Prior distributions 18
6.5.3 Computational details 19
6.6 Model outputs and diagnostics 19
6.6.1 Figure e2: trace plots for length-constant ρ 20
6.7 Clinical validity 20
6.7.1 Correlation between variables 21
6.7.2 Figure e3: correlation matrix across parameters – MIMIC 21
6.7.3 Future-held-out validation 22
6.7.4 Table e3: Future-held-out validation of confounder model, continuous variables 23
6.7.5 Table e4: Future-held-out validation of confounder model, binary variables 24
6.7.6 Figure e4: Example validation trajectory 25
7 Conditional outcome model 26
7.1 Bayesian additive regression trees in causal inference 26
7.2 Model description and computational details 26
7.3 Covariates 27
7.4 Model outputs and diagnostics 27
7.5 Five-fold cross-validation 28
7.5.1 Figure e5: Calibration of conditional outcome model 29
7.5.2 Figure e6: Discrimination and precision of conditional outcome model – MIMIC-IV 30
7.5.3 Figure e7: Discrimination and precision of conditional outcome model – AmsterdamUMCdb 30
8 Monte Carlo integration 31
8.1 Modeled usual care 31
8.2 Treatment thresholds and death before invasive ventilation during the target trial period 31
8.3 Simulated trajectories 32
8.4 Figure e8: example simulated trajectory 33
9 Additional results 34
9.1 Relative risks and e-values 34
10 Additional tables and figures 35
10.1 Table e5: Comparison of MIMIC-IV and AmsterdamUMCdb cohorts and analyses 35
10.2 Figure e9: Target trial eligibility flow diagrams 36
11 References 37

1 STROBE statement

STROBE Statement—Checklist of items that should be included in reports of ***cohort studies***

	Item No	Recommendation
**Title and abstract**	1	(*a*) Indicate the study's design with a commonly used term in the title or the abstract **(DONE, p1, p6)**
		(*b*) Provide in the abstract an informative and balanced summary of what was done and what was found **(DONE, p6)**
**Introduction**		
Background/rationale	2	Explain the scientific background and rationale for the investigation being reported **(DONE, p8-9)**
Objectives	3	State specific objectives, including any prespecified hypotheses **(DONE, p9)**
**Methods**		
Study design	4	Present key elements of study design early in the paper **(DONE p9-14)**
Setting	5	Describe the setting, locations, and relevant dates, including periods of recruitment, exposure, follow-up, and data collection **(DONE p9-12)**
Participants	6	(*a*) Give the eligibility criteria, and the sources and methods of selection of participants. Describe methods of follow-up **(DONE p10)**
		(*b*) For matched studies, give matching criteria and number of exposed and unexposed **N/A**
Variables	7	Clearly define all outcomes, exposures, predictors, potential confounders, and effect modifiers. **P9-11, supplement section 4**
Data sources/ measurement	8*	For each variable of interest, give sources of data and details of methods of assessment (measurement). Describe comparability of assessment methods if there is more than one group **(DONE p9-11 and supplement section 4)**
Bias	9	Describe any efforts to address potential sources of bias **DONE p10-13,** **supplement sections 6-9**
Study size	10	Explain how the study size was arrived at **DONE p9-10**
Quantitative variables	11	Explain how quantitative variables were handled in the analyses. If applicable, describe which groupings were chosen and why **supplement section 4 and section 7.5**
Statistical methods	12	(*a*) Describe all statistical methods, including those used to control for confounding **DONE p11-13 and supplement sections 6 – 9**
		(*b*) Describe any methods used to examine subgroups and interactions **p12**
		(*c*) Explain how missing data were addressed **DONE p11-14**
		(*d*) If applicable, explain how loss to follow-up was addressed **N/A**
		(*e*) Describe any sensitivity analyses **DONE p13-14**
**Results**		

Participants	13*	(a) Report numbers of individuals at each stage of study—eg numbers potentially eligible, examined for eligibility, confirmed eligible, included in the study, completing follow-up, and analysed **DONE Figure e9**
		(b) Give reasons for non-participation at each stage **Figure e9**
		(c) Consider use of a flow diagram **Figure e9**
Descriptive data	14*	(a) Give characteristics of study participants (eg demographic, clinical, social) and information on exposures and potential confounders **Table 1 Table e4**
		(b) Indicate number of participants with missing data for each variable of interest **N/A**
		(c) Summarise follow-up time (eg, average and total amount) **N/A**
Outcome data	15*	Report numbers of outcome events or summary measures over time **p13 p15**
Main results	16	(*a*) Give unadjusted estimates and, if applicable, confounder-adjusted estimates and their precision (eg, 95% confidence interval). Make clear which confounders were adjusted for and why they were included **14-17**
		(*b*) Report category boundaries when continuous variables were categorized **supplement p11-17**
		(*c*) If relevant, consider translating estimates of relative risk into absolute risk for a meaningful time period **DONE, absolute risk / RR / OR reported**
Other analyses	17	Report other analyses done—eg analyses of subgroups and interactions, and sensitivity analyses **p14 p 17**
**Discussion**		
Key results	18	Summarise key results with reference to study objectives **p17**
Limitations	19	Discuss limitations of the study, taking into account sources of potential bias or imprecision. Discuss both direction and magnitude of any potential bias **p17-20**
Interpretation	20	Give a cautious overall interpretation of results considering objectives, limitations, multiplicity of analyses, results from similar studies, and other relevant evidence **p19-20**
Generalisability	21	Discuss the generalisability (external validity) of the study results **p17-20**
**Other information**		
Funding	22	Give the source of funding and the role of the funders for the present study and, if applicable, for the original study on which the present article is based **p4**

2 Target trial details: Secondary thresholds

The primary thresholds focused on oxygenation via the saturation-to-inspired oxygen ratio (SF). We also considered additional thresholds to capture other important dimensions of respiratory failure.

2.1.1 Respiratory rate

Respiratory rate is a common element in criteria for invasive ventilation found in observational (1–3) and randomized (4–6) studies. We included invasive ventilation thresholds with the same degree of hypoxemia (SF ratio of less than 98) and different degrees of tachpynea (respiratory rate of 25 breaths per minute or more versus 35 breaths per minute or more).

2.1.2 Work of breathing

Increased work of breathing is commonly mentioned in qualitative studies of the criteria for invasive ventilation.(7,8) We included thresholds of an SF ratio less than 98 with and without abnormal work of breathing. This variable was only available in the MIMIC cohort.

2.1.3 Duration

Observational research has found that a failure to improve from a certain degree of severity is associated with subsequent invasive ventilation.(2,9) We investigated the importance of duration with thresholds requiring an SF ratio less than 98 for one measurement, for two consecutive hours, and for four consecutive hours.

2.1.4 Trajectory

One motivation for the use of invasive ventilation is to avoid a more dangerous deterioration if the current clinical trajectory were to continue. We used linear extrapolation of the current and immediately prior SF ratios to predict the next SF ratio and included both a moderate trajectory (SF ratio predicted to be less than 88 within 60 minutes) and a severe trajectory (SF ratio predicted to be less than 88 within 30 minutes).

2.1.5 Multi-organ involvement

The criteria for invasive ventilation used in many trials of respiratory support include elements of respiratory, neurologic, and hemodynamic function.(5) Criteria are usually satisfied by dysfunction in any one of the three areas. We included a threshold focused on single organ failure (criteria met for any of those three areas) and a threshold requiring dual-organ failure (respiratory and either neurologic or hemodynamic criteria met). The organ system-based criteria were as follows: respiratory criteria = 2 of RR > 40, saturation < 90 on inspired oxygen 0.90 or higher, abnormal work of breathing, or pH < 7.35; hemodynamic criteria = use of vasopressors; neurologic criteria = Glasgow Coma Scale < 9

2.1.6 Usual care

In order to compare the outcomes of each threshold to usual care, we also included a threshold based on usual care. This was done by incorporating invasive ventilation as a binary variable into the confounder model, which allowed for us to predict at each clinical measurement whether or not a patient was likely to be invasively ventilated before the next measurement. Those predictions were incorporated in the Monte Carlo integration as described in section 10.1 to allow for estimation of the probability of invasive ventilation and mortality under usual care, using the exact same clinical trajectories across which all the other thresholds were compared.

2.2 Table e1: Target trial characteristics

<table>
<tr><th></th><th>Target trial specification</th><th>Target trial emulation</th></tr>
<tr><td>Eligibility criteria</td><td>Inclusion
• Age 18 years or older
• Admission to ICU within prior 24 hours
• Receiving oxygen via non-rebreather mask, high-flow nasal cannula, or non-invasive ventilation with an inspired oxygen fraction of 0.4 or greater
Exclusion
• GCS motor score (< 4), respiratory acidosis (pH ≤ 7.20 with pCO_2 ≥ 60), or clinical judgement
• Care limitations restricting use of invasive ventilation
• Prior use of invasive ventilation during same ICU admission
• Admitted to ICU directly from operating room
• Tracheostomy in situ
• Prior eligible ICU admission</td><td>Same, except:
• No ability to incorporate subjective clinical judgement of a lack of equipoise</td></tr>
<tr><td>Time zero</td><td>Treatment assignment</td><td>Eligibility criteria satisfied</td></tr>
<tr><td>Treatment strategies</td><td>Each strategy is a threshold which, if met, prompts intubation and invasive ventilation within 1 hour by the clinical team. Thresholds are active for 96 hours after which usual care prevails.

Hypoxemia:
• SF ratio less than 88
• SF ratio less than 98
• SF ratio less than 110

Tachypnea:
• SF ratio less than 98 and respiratory rate greater than 25
• SF ratio less than 98 and respiratory rate greater than 35

Abnormal work of breathing:
• SF ratio less than 98 and abnormal work of breathing

Duration:
• SF ratio less than 98 for 2 consecutive hours
• SF ratio less than 98 for 4 consecutive hours

Trajectory: based on linear extrapolation of the SF ratio
• SF ratio less than 88 in 60 minutes
• SF ratio less than 88 in 30 minutes

Multi-organ involvement: based on randomized trial criteria*
• Respiratory, neurologic, or hemodynamic failure
• Respiratory and either neurologic or hemodynamic failure</td><td>Same, except:
• Immediate invasive ventilation
• No protocol deviation possible</td></tr>
<tr><td>Treatment assignment</td><td>Each patient randomized to one threshold</td><td>Each threshold is applied to every patient in sequence. Treatment assignment will be considered “at random” conditional on measured confounders. Bayesian modeling used to ensure appropriate quantification of uncertainty</td></tr>
<tr><td>Outcomes</td><td>Mortality at 28 days</td><td>Same</td></tr>
<tr><td>Follow-up</td><td>Starts at baseline and continues up to 28 days</td><td>Same</td></tr>
<tr><td>Contrasts</td><td>Intention-to-treat, per-protocol</td><td>Per-protocol</td></tr>
</table>

3 Cohort construction – MIMIC-IV

Cohort construction was carried out in a similar fashion to our complementary investigation of whether patients receive invasive ventilation after they meet physiologic thresholds.(10) As a result, the text below is intentionally similar to the text describing cohort construction in the supplement of that manuscript. The names of tables and variables from the datasets will be written in italics. Where specific identifying numbers (eg *itemid*) were used in the MIMIC cohort we include them here, to facilitate transparency.

3.1 Eligibility assessment

Eligibility assessment required knowledge of a patient's oxygen device and their fraction of inspired oxygen at the same moment. Both MIMIC-IV and AmsterdamUMCdb data include data points timestamped to (at least) the nearest minute, which introduces irregular sampling. The observations of oxygen device and fraction of inspired oxygen may not be concurrent. We carried forward observations of fraction of inspired oxygen and oxygen device for up to 8 hours.(15) The patient was deemed eligible at the first time within the first 24 hours of ICU admission where the fraction of inspired oxygen was 40% or more while using a non-rebreather mask, high flow nasal cannula, or non-invasive ventilation.

3.2 Specific variables

Below we outline the decisions made to gather specific variables from the MIMIC-IV data tables.

3.2.1 Tracheostomies

Tracheostomies were identified by an oxygen device charting of "Tracheostomy tube" or "Trach mask". Any patient with a tracheostomy charted within the first 144 hours following ICU admission was excluded from the study.

3.2.2 Goals of care

Goals of care were identified from *chart events* (*itemid 228687*) where clinicians recorded updates about the goals of care. Patients with “"Comfort measures only" or "DNAR (Do Not Attempt Resuscitation) [DNR] / DNI"

charted at any time during their admission were excluded. However, very few patients had anything recorded under this variable. The clinical notes were not available. The lack of better prospective information on goals of care likely made it more difficult for the confounder model to predict invasive ventilation or death (before invasive ventilation), and remains a limitation of the study. However, the unintentional inclusion of patients who were “randomized” by their goals of care to a “never” strategy of invasive ventilation provides the confounder model with information about what happens to patients who do not undergo invasive ventilation.

3.2.3 Race/ethnicity

The eight race/ethnicity categories (“WHITE”, “UNKNOWN”. “BLACK/AFRICAN AMERICAN”, “ASIAN”, “HISPANIC/LATINO”, “OTHER”, “UNABLE TO OBTAIN”, “AMERICAN INDIAN/ALASKA NATIVE.”) were collapsed into six categories because the American Indian / Alaska Native category had too few patients to report, in the spirit of deidentification. The six categories were White, Black, Asian, Hispanic, Other (including American Indian / Alaska Native) and Unknown.

3.2.4 Care unit

The 9 potential care units ("Coronary Care Unit (CCU)”, "Medical Intensive Care Unit (MICU)”, "Surgical Intensive Care Unit (SICU)", "Trauma SICU (TSICU)", "Medical/Surgical Intensive Care Unit (MICU/SICU)", "Cardiac Vascular Intensive Care Unit (CVICU)", "Neuro Surgical Intensive Care Unit (Neuro SICU)", "Neuro Stepdown", "Neuro Intermediate") were collapsed into three: Medical-Surgical, Cardiac, and Neuro-Trauma.

3.2.5 Fraction of inspired oxygen

Fraction of inspired oxygen was taken directly from the corresponding field where available. If no charted fraction of inspired oxygen was available, and the patient was receiving oxygen by non-rebreather mask, face mask, or nasal prongs, then the oxygen flow was used to estimate the fraction of inspired oxygen by a validated equation(16): 21% + oxygen flow rate in L/min*3.

3.2.6 Work of breathing

The work of breathing variable was composed from different fields relating to the pattern of respiration (chart event IDs 229322, 223990, 229323). Patterns described as 'Dyspneic', 'Labored', 'Shallow', 'Apneic', 'Agonal', 'Discoordinate', 'Gasping efforts', 'Prolonged exhalation', 'Shallow', 'Irregular', 'Nasal flaring', 'Cheyne-Stokes', 'Accessory muscle use/retractions', 'Frequent desaturation episodes', 'Inability to speak in full sentences', and 'Active exhalation' were classified as abnormal. Normal observations included 'Regular' or 'Normal'. The chart event ID 229323 was labeled the "Current Dyspnea Assessment" and recorded dyspnea on a scale from 0 to 10. Dyspnea levels of 'Moderate - 4', 'Moderate - 5', 'Moderate - 6', 'Moderate - 7', 'Severe - 8', 'Severe - 9', or 'Severe - 10' were classified as abnormal, while dyspnea levels of 'None - 0', 'Mild - 1', 'Mild - 2', or 'Mild - 3' were classified as normal.

4 Cohort construction – AmsterdamUMCdb

4.1 Eligibility assessment

This was done in the same manner as for MIMIC-IV. Here we comment on differences.

4.2 Validated observations

The AmsterdamUMCdb cohort includes a large number of observations gathered automatically from the monitors and the ventilators. For every observation, there is a field describing if the value was "registered" or validated by a clinician. As in the code generated in datathons focusing on the AmsterdamUMCdb data (https://github.com/AmsterdamUMC/AmsterdamUMCdb), we opted to include only validated data in the cohort. There were two reasons (1) better assurance of its veracity and (2) without filtering, the data was too voluminous with observations occurring as frequently as every minute for certain variables.

4.3 Specific variables

4.3.1 Table e2: Oxygen devices

We used the following translations for oxygen devices:

	Category	ID	Dutch	English
8189	Toedieningsweg	1	Diep Nasaal	Nasal oxygen catheter (ie for nasal suctioning)
8189	Toedieningsweg	2	Nasaal	Nasal oxygen cannula (low-flow)
8189	Toedieningsweg	3	Kapje	Venturi mask
8189	Toedieningsweg	4	Kunstneus	Heat- and moisture exchanger that you connect to the tracheostomy or tube when the patient is disconnected from the ventilator
8189	Toedieningsweg	7	O2-bril	Nasal oxygen cannula (low-flow)
8189	Toedieningsweg	8	Kinnebak	Face tent

8189	Toedieningsweg	9	Nebulizer	Nebulizer
8189	Toedieningsweg	10	Waterset	Bag-valve mask
8189	Toedieningsweg	11	Trach.stoma	Tracheal stoma
8189	Toedieningsweg	12	B.Lucht	Room air
8189	Toedieningsweg	13	Ambu	Ambubag
8189	Toedieningsweg	14	Guedel	Oropharyngeal airway
8189	Toedieningsweg	15	DL-tube	Double-lumen tube
8189	Toedieningsweg	16	CPAP	CPAP mask
8189	Toedieningsweg	17	Non-Rebreathing masker	Non-rebreather mask
8189	Toedieningsweg	18	Spreekcanule	Speaking valve for tracheostomy

4.3.2 Goals of care

Goals of care were encoded under a listitems item called "Beleid" ("policy"). There were three values: 1 (no restrictions), 2 (restrictions such as no dialysis, no CPR, no ventilation), and 3 (no escalation / terminal). For many patients there was no "Beleid" observation available. In keeping with goals of care as an exclusion criteria for the study (as opposed to an inclusion criteria), we excluded patients with goals of care type 3.

4.3.3 Operative

Patients with operative APACHE diagnoses were excluded.

4.3.4 Invasive ventilation

Invasive ventilation start times were identified by the earliest of (1) charting of an intubation procedure in the procedureorderitems table or (2) start time of an episode of invasive ventilation from the processitems table.

4.3.5 Care unit

Patients were cared for in either the intensive care unit (ICU) or the MCU, which was a "step-down" level unit.

5 Using the G-formula in this study: rationale and explanation

There are two primary analytic options for evaluating prespecified dynamic treatment regimens in the setting of time-varying confounding: inverse probability weighting with clones and g-computation.(12) By dynamic treatment regimen, we mean a sequential treatment assignment that follows a pre-determined function of baseline and time-varying patient information. For this study, we selected G-computation in order to use a Bayesian approach with a prespecified observation schedule. The G-formula above breaks into two parts, the confounder model and the conditional outcome model. To mitigate the possibility of model misspecification, we used non-parametric Bayesian models for both the conditional outcome and time-varying confounder models.(13) We will describe both models in detail, starting with the confounder model. All code is available at https://doi.org/10.5281/zenodo.7314132.

6 Confounder model

The confounder model aims to describe the variables that change over time and are associated with both the use of invasive ventilation and mortality. Baseline confounders are addressed with the conditional outcome model. Using domain expertise and prior research, we selected several physiologic variables as confounders (see Figure).(1–3,14–16) We selected respiratory rate, peripheral oxygen saturation, heart rate, systolic blood pressure, fraction of inspired oxygen, Glasgow Coma Scale, lactate, partial pressure of carbon dioxide, pH, vasopressor use, and oxygen device. For the MIMIC cohort we included abnormal work of breathing as an additional covariate, and for the AmsterdamUMCdb cohort we included partial pressure of arterial oxygen. For both cohorts we also incorporated binary variables of ICU discharge, invasive ventilation, and death before invasive ventilation. For the confounder model we used a multitask Gaussian process with a linear covariance structure because it is one of the few models that is generative, flexible, and can output multiple correlated time series variables.

6.1 Figure e1: Conceptual model

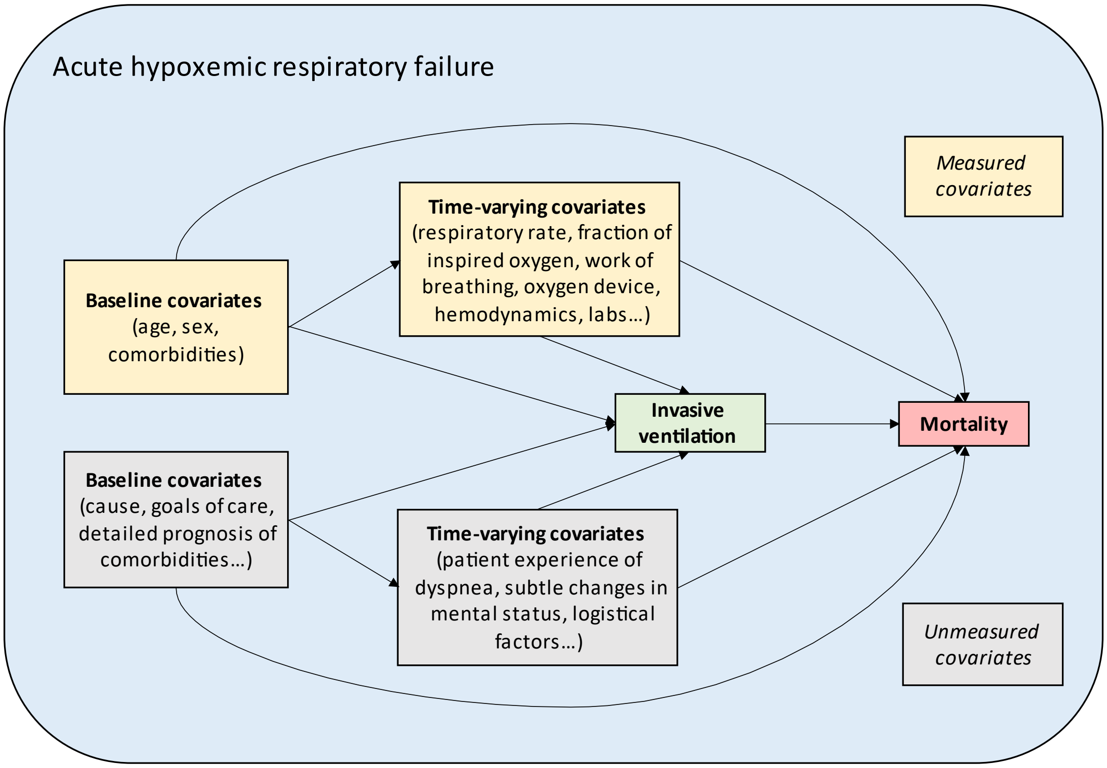

6.2 Multitask Gaussian processes in clinical medicine

Gaussian processes are a class of model well-suited to describing multivariable clinical time series, because they easily accommodate irregularly sampled data, learn correlations between variables, and quantify the extent to which prior observations influence future observations. They are scientifically appealing because each particular realization of a Gaussian process is stochastic, analogous to each individual patient's multivariable trajectory in the ICU, but every realization is bound by the same underlying distribution of covariance between variables and across time, analogous to the underlying physiologic process of critical illness. They allow for prior information to guide the timescales over which past variables influence future variables, but they do not require arbitrary decisions such as restricting variables to linear relationships, or aggregating data into discrete time points. For further details and introduction to Gaussian process modeling, we suggest the Bayesian Data Analysis textbook by Gelman et al and the blog posts of Betancourt.(17,18)

6.3 Hilbert-space Gaussian process approximation

A pure Gaussian process has a computational time that scales with the cube of the number of observations (N^3). For our dataset with hundreds of thousands of observations, this is impractical. Fortunately, Bayesian biostatisticians Riutort-Mayol and Vehtari have described an accurate Hilbert space approximation to Gaussian processes.(19) We used this approximation.

The Hilbert space approximation relies on specification of an appropriate boundary constant C and number of basis functions M. The choice depends on the underlying length constant ρ of the Gaussian process that best fits the data and the type of Gaussian process covariance function chosen. We chose a squared exponential covariance function, boundary conditions $C = 1.25$ and $M = 20$, which meant that the smallest length constant ρ (normalized by the half-range of the data) that we could estimate was 0.11. As long as our estimated length constant was larger, the approximation will be accurate to the fit provided by a true Gaussian process.

6.4 Data transformations

In order to facilitate model fitting, we performed some basic data transformations. Heart rate, respiratory rate, systolic blood pressure, lactate, partial pressure of carbon dioxide, partial pressure of arterial oxygen, and pH were all transformed with natural logarithms. Fraction of inspired oxygen, saturation, and Glasgow Coma Score were all treated as continuous interval data and transformed with the inverse logit function parameterized according to the interval of each variable.(20,21) After transformation, all of the above continuous variables were centered at the transformed mean and scaled by the transformed standard deviation. Time was transformed to days as a continuous variable.

To better approximate the clinical reality that some variables are only recorded when they change, we filled in measurements of inspired oxygen fraction, oxygen device, vasopressor use, ICU discharge status, invasive ventilation status, and death status every 2 hours in addition to the measurements already present in the data. This allowed for the inclusion of these variables in the Gaussian process.

6.5 Model description

We used a Gaussian process to fit the function $\mu(t) = \vec{y}$ for time t and multivariable clinical time series $\vec{y} \in R^d$. For the confounder model, $d = 16$ (AmsterdamUMCdb) or 17 (MIMIC-IV). (heart rate, respiratory rate, systolic blood pressure, mean blood pressure, peripheral oxygen saturation, fraction of inspired oxygen, partial pressure of carbon dioxide, partial pressure of arterial oxygen, pH, GCS, non-invasive ventilation use, non-rebreather mask use, invasive ventilation, ICU discharge, and death for both cohorts; partial pressure of arterial oxygen for AmsterdamUMCdb, and abnormal work of breathing and high-flow nasal cannula use for MIMIC-IV). The components of $\vec{y}$ were the latent variables underlying the observed clinical variables. For continuous variables, the observed variable differs from the latent variable by independent identically distributed random noise with standard deviation σ. For binary variables, the observed variable is a random binary variable (0 or 1) with underlying probability given by the latent variable including its offset via the inverse logit transformation. This innovation allows for covariance between binary and continuous variables.

A Gaussian process $\mu \sim \mathrm{GP}(m, k)$ is specified by the mean function m and covariance function k. We used the squared exponential covariance function:

$$\kappa(t_1, t_2) = \alpha e^{\frac{-(t_1 - t_2)^2}{\rho}}$$

This covariance function generates smooth curves with “wiggliness” based on the length-scale ρ and height based on the magnitude α. The length-scale ρ dictates the time over which the Gaussian process “forgets” prior values. We assumed that the hyperparameters ρ and α were constant over all patients in the cohort and over the entire observation time of up to 96 hours.

6.5.1 Mean function

We used a time-invariant mean function for each latent variable. We included eleven covariates via linear regression for each latent variable. This included five categories of age, sex, and then five additional baseline

variables which usually corresponded to the five quintiles of the baseline value for the latent variable in question. For example, the mean heart rate for a given patient was a linear function of their age, sex, and the quintile of their baseline heart rate. The covariates were selected using clinical domain expertise.

6.5.2 Prior distributions

While flexibility is a strength of Gaussian processes, it can also doom computational efforts to fit the model unless domain expertise is used to supply informative priors for the hyperparameters ρ and α. The length-scale in particular requires informed prior distributions, else any efforts to fit the model will tend towards either a length scale of infinity (the function is equal to the mean function at every point with the addition of uncorrelated noise) and a length-scale of zero (the function is infinitely wiggly with no memory). Based on the work of Cheng et al with MIMIC-III data, we chose a lognormal prior centered at approximately $e^{-1.5} = 0.22$ with standard deviation 0.2 that amounted to a 95% probability of the timescale being between 7 and 16 hours.(22)

For the magnitudes, we used less informed lognormal priors with standard deviations of 1 for continuous variables and 2 for binary variables. The means of the magnitudes, noise, and offsets were placed near the final estimated means using a trial run on a subset of the data, because the speed of fitting the model on the entire dataset was running up against the 24-hour time limit imposed by our computer cluster. This introduces an empirical Bayesian element into the model, and may cause us to overestimate the certainty of our results. However, when the model was run with prior means uninformed by the data on smaller subsets of the cohort, there were no difficulties in fitting the model and the results matched. The prior standard deviation for the noise was 1 on the lognormal scale. The prior standard deviation for the offsets was set at 10.

The Hilbert space Gaussian process approximation uses basis functions that are specific to each patient and variable. The prior distributions for these basis functions were set to standard normal distributions.(19) The

mean function covariates had normal distributions centered at 0 with standard deviation 0.5 as priors. For the linear covariance matrix prior, we used the Cholesky decomposition of the LKJ(3) matrix.

6.5.3 Computational details

We used Hamiltonian Monte Carlo sampling via Stan to fit the Hilbert space Gaussian process approximation.(23) The program was written in Stan and is available at https://doi.org/10.5281/zenodo.7314132. The program was optimized for faster fitting by (1) use of the *reduce_sum* function allowing for within-chain parallelization and (2) extensive use of the offset and multiplier functionality when declaring parameters. This had the unfortunate consequence of reducing code readability.

In order to fit the model we used the Compute Canada cluster, parallelizing each chain over 80 cores. The Compute Canada cluster has a maximum runtime of 24 hours. In order to achieve convergence and sampling within 24 hours on the largest possible sample size, we had to initialize chains to the center of their prior distributions. Chains initialized randomly required longer warmup runs in order to sample effectively, and did achieve convergence when run on smaller subsets of the population (eg 400 patients instead of 1100-1200). We ran 200 iterations warmup and 200 iterations sampling per chain, 4 separate chains for each model fit. We did not need to increase the target acceptance rate (adapt_delta) parameter above the default of 0.8.

6.6 Model outputs and diagnostics

For the MIMIC-IV cohort, we had to split the sample randomly into three folds of approximately 1100 patients each and run the confounder model separately on each fold. With this approach, each chain parallelized across 80 cores from one node took approximately 20 hours to warmup and sample 200 iterations. For the AmsterdamUMCdb cohort we were able to run the model on the entire 1279 person cohort in 22-24 hours per chain. Therefore we used 3*4*20*80 + 4*22*80 = 26,240 core-hours to generate 800 samples per cohort.

For all chains in all folds, there were no divergent transitions and the estimated Bayesian fraction of missing information was low. A subset of basis function parameters (*eta*) had a low effective sample size. This is not

surprising because if a patient has no or few measurements of a given variable, the corresponding basis functions will have minimal information beyond the standard normal distribution prior. A subset of parameters also had an R-hat value of greater than 1.05, suggesting incomplete mixing. Ideally, we would have been able to run more samples to address this issue, but fortunately we also used future-held-out validation to evaluate the confounder model fit as well. We did not have access to sufficient computing resources to run more samples from the model.

The trace plots generally sufficient mixing, with some variables showing a bit more autocorrelation than would be ideal. Some examples shown below.

6.6.1 Figure e2: trace plots for length-constant ρ

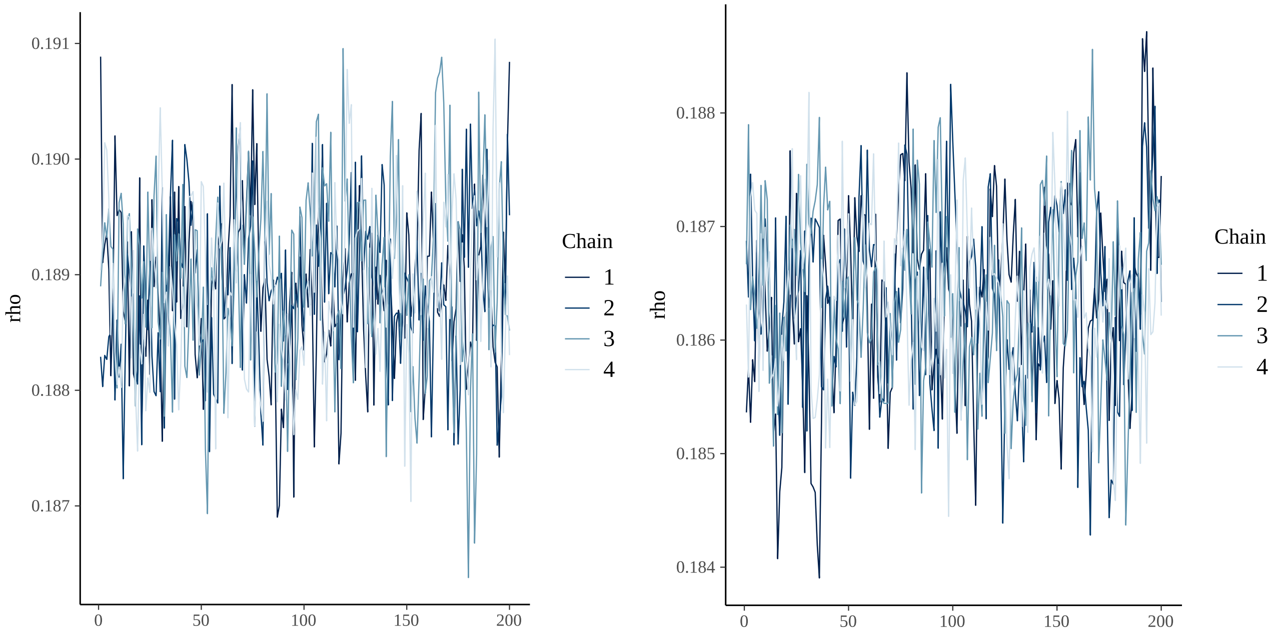

Caption: This figure shows the trace of the posterior samples of the length constant for MIMIC-IV analysis (left) and AmsterdamUMCdb analysis (right).

6.7 Clinical validity

In this study, the confounder model has two (related) roles: first, to describe the time-varying confounders between exposure and outcome, and second, to generate realistic clinical trajectories for the integration required to calculate the G-formula. The validity of the final results depends in part on the validity of the

confounder model. Therefore, we investigated the predictive validity of the confounder model through quantitative and qualitative means.

6.7.1 Correlation between variables

First, we inspected the correlation matrices of the confounder model. These showed that the Gaussian process, without any prior information about the relationships between variables (we used a skeptical LKJ(3) prior), was able to identify clinically relevant correlations. For example, respiratory rate is correlated with inspired oxygen fraction and inversely correlated with peripheral oxygen saturation in both cohorts. The MIMIC-IV correlation matrix is shown as an example.

6.7.2 Figure e3: correlation matrix across parameters – MIMIC

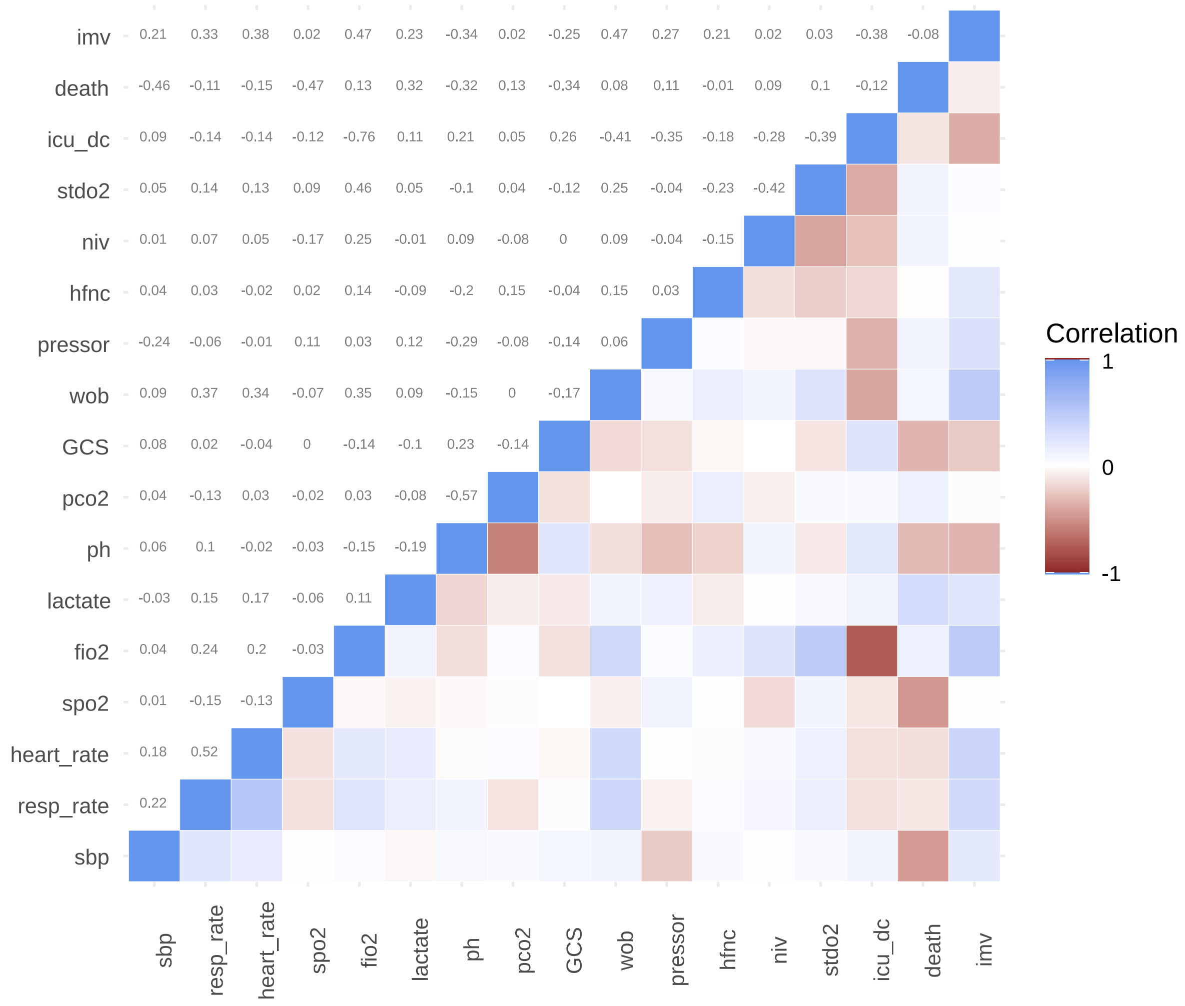

Caption: This figure shows the correlation matrix between the posterior parameters for all of the time-varying confounding variables. Correlated pairs are marked with blue, anticorrelated with red, and no correlation with white.

6.7.3 Future-held-out validation

We performed future-held-out validation on the same time scale as our proposed target trial observation schedule. To do this, we divided the timeline into two-hour time steps from 0 hours to 92 hours. We then used the means of the posterior distribution of Gaussian process hyperparameters to fit a Gaussian process to all data available up to the start of a time step. We used the means instead of individual hyperparameter draws because the posterior distributions of hyperparameters were very tight and it was not computationally feasible to validate all time steps for all posterior hyperparameter values. We then used that Gaussian process to predict all observations that happened between 1 and 3 hours after the start of that time step. For example, we fit a Gaussian process using all data available up until time = 10 hours and then predicted the values of all observations made between time = 11 hours and time = 13 hours. Recall that the observation schedule uses measurements every 2 hours. For computational reasons, we were able to validate on only 400 randomly selected patients. This allowed us to compare predictions of both continuous and binary covariates from the confounder model to the actual measured values.

Below we show tables of root-mean-squared error, discrimination, and precision of the variables from each confounder model. We also show an example validation trajectory for a single patient, chosen at random from the MIMIC-IV cohort.

6.7.4 Table e3: Future-held-out validation of confounder model, continuous variables

Variable	Observed mean	Posterior mean	RMSE	Coverage (95%)
**MIMIC-IV**				
Systolic blood pressure	118.51	117.81 (115.15 to 121)	20.87 (18.93 to 23.84)	0.94 (0.84 to 0.98)
Respiratory rate	22.63	21.77 (21.07 to 23.35)	6.87 (6.13 to 7.77)	0.94 (0.87 to 0.97)
Heart rate	94.16	90.23 (88.33 to 96.07)	14.38 (12.36 to 19.32)	0.95 (0.86 to 0.97)
Peripheral saturation	95.74	94.93 (94.42 to 95.51)	5.07 (4.32 to 6.67)	0.92 (0.84 to 1)
Inspired oxygen fraction	58.1	50.42 (45.97 to 62.65)	18.2 (14.96 to 26.18)	0.93 (0.74 to 0.97)
Lactate	2.09	2.11 (1.08 to 3.05)	1.2 (0.14 to 2.35)	0.66 (0 to 1)
pH	7.38	7.38 (7.33 to 7.44)	0.08 (0.04 to 0.12)	0.82 (0 to 1)
pCO2	45.05	47.58 (38.7 to 59.8)	13.5 (4.62 to 23.26)	0.79 (0 to 1)
GCS	13.55	14.23 (13.86 to 14.66)	1.76 (0.81 to 2.57)	0.95 (0.89 to 1)
**AmsterdamUMCdb**				
Systolic blood pressure	126.23	132.72 (125.37 to 140.6)	27.6 (23.69 to 34.83)	0.94 (0.8 to 0.99)
Respiratory rate	25.57	23.53 (21.57 to 26.36)	9.79 (7.66 to 12.15)	0.92 (0.81 to 0.99)
Heart rate	102.05	93.87 (89.97 to 101.2)	15.06 (11.36 to 24.02)	0.93 (0.7 to 0.99)
Peripheral saturation	95.95	95.82 (95.17 to 96.54)	4.25 (3.12 to 5.85)	0.93 (0.82 to 1)
Inspired oxygen fraction	57.76	55.09 (50.67 to 59.81)	14.37 (10.68 to 18)	0.95 (0.84 to 1)
Lactate	2.27	1.55 (0.8 to 2.42)	0.87 (0.18 to 1.87)	0.77 (0 to 1)
pH	7.36	7.41 (7.37 to 7.44)	0.07 (0.04 to 0.1)	0.94 (0.75 to 1)
pCO2	41.95	42.17 (38.71 to 45.76)	9.8 (6 to 15.2)	0.89 (0.62 to 1)
GCS	13.55	13.76 (12.43 to 14.74)	2.23 (0.67 to 5.52)	0.83 (0 to 1)
pO2	112.39	92.53 (81.48 to 103.9)	37.62 (23.5 to 54.19)	0.94 (0.77 to 1)

6.7.5 Table e4: Future-held-out validation of confounder model, binary variables

Variable	Observed mean probability	Predicted mean probability	Discrimination (AUROC)	Precision
**MIMIC-IV**				
Vasopressor use	0.1565	0.1228	0.903	0.584
Non-invasive ventilation	0.0442	0.0507	0.891	0.305
High-flow nasal cannula	0.1761	0.1679	0.876	0.638
Non-rebreather mask	0.3001	0.3334	0.817	0.637
Abnormal work of breathing	0.2301	0.2462	0.822	0.582
ICU discharge	0.0232	0.0200	0.686	0.072
Death before IMV	0.0018	0.0013	0.779	0.092
Invasive ventilation	0.0079	0.0069	0.678	0.027
**AmsterdamUMCdb**				
Vasopressor use	0.1933	0.1608	0.950	0.857
Non-invasive ventilation	0.1280	0.1323	0.939	0.798
Non-rebreather mask	0.3372	0.3664	0.947	0.876
ICU discharge	0.0247	0.0226	0.688	0.053
Death before IMV	0.0021	0.0013	0.929	0.072
Invasive ventilation	0.0108	0.0095	0.737	0.029

ICU = Intensive care unit. IMV = invasive mechanical ventilation. AUROC = area under receiver-operating curve. PPV = positive predictive value. The observed and predicted means are similar, with the largest relative differences coming in the rare binary events of discharge, death, and invasive ventilation. The discrimination is very good for the frequently measured clinical variables (vasopressor use, oxygen devices, work of breathing). The precision is also good for the same variables, except for non-invasive ventilation use in MIMIC which has a lower precision. Among the binary event variables, the precision is very low. This likely reflects the difficulty of pinpointing a time at which each of those events will happen, in addition to the unmeasured non-physiologic confounding that impacts the timing of each transition. This non-physiologic confounding could be anything from unavailability of a bed on the ward for transfer to a sudden shift in goals of care.

6.7.6 Figure e4: Example validation trajectory

MIMIC

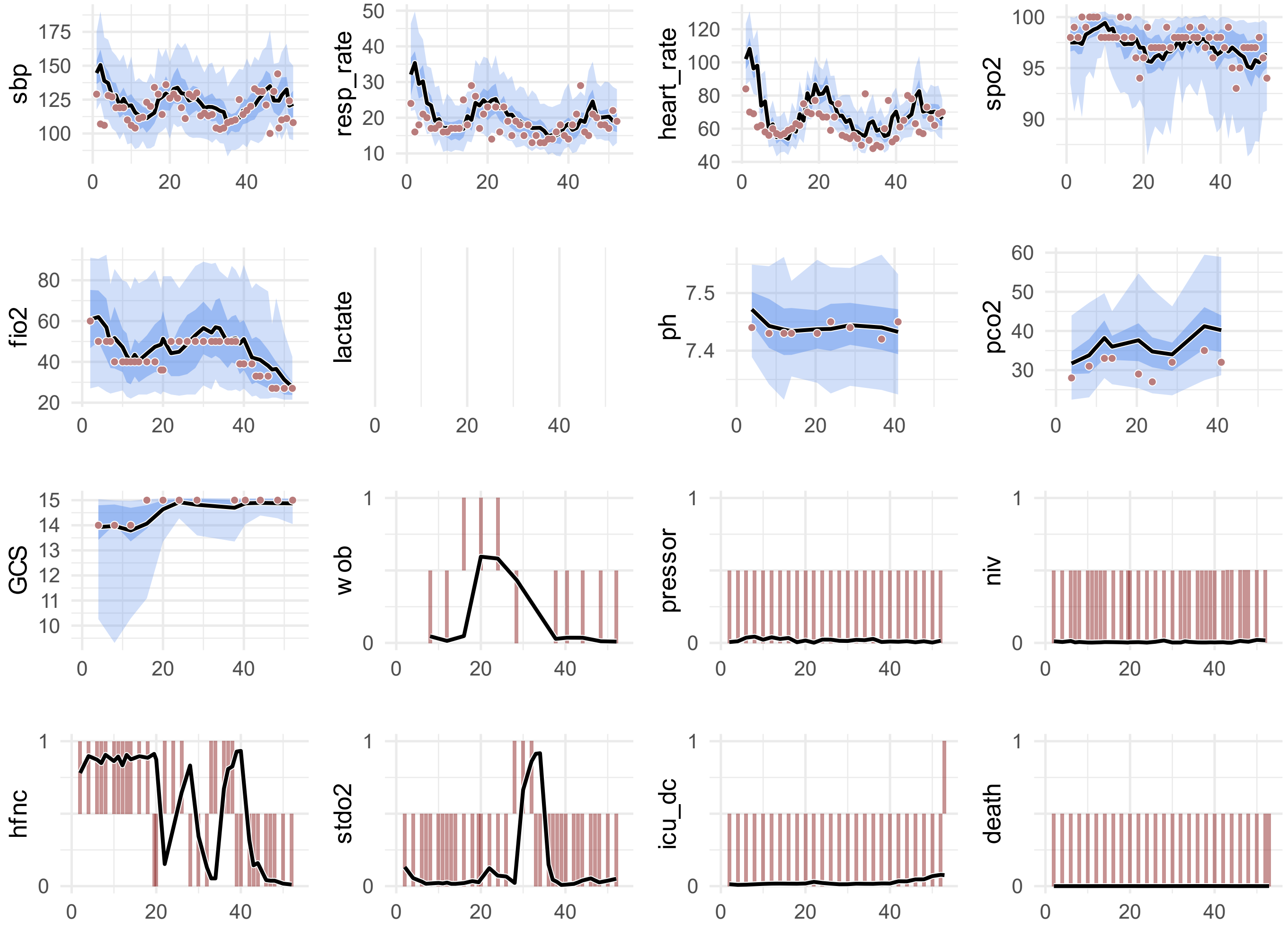

Caption: This validation trajectory shows the futre-held-out observed data (red) and the predictions (mean = black, 50% credible interval = dark blue, 95% credible interval = light blue). The binary variables show only the underlying mean probability of an event (black). The data used for each prediction ends between 1 and 3 hours before each prediction, approximating the situation of the q2h observation schedule in the target trial. The figure shows both the strengths and weaknesses of the model. Strengths include the ability to follow the arbitrary curves of continuous variables, the state-switching of the binary variables, and appropriate communication of uncertainty where data is sparse (eg blood gas results). The weaknesses are that the mean does not vary with time (see slight tendency towards overestimation of respiratory rate and inspired oxygen fraction) and that rare binary events are difficult to predict (see icu_dc variable).

7 Conditional outcome model

The conditional outcome model is used to predict the mortality of each patient after observing their target trial emulation time series. As a reminder, that time series ends after the patient reaches the end of the 96 hours without being invasively ventilated, or when the patient is no longer eligible for the treatment rule (death before invasive ventilation, ICU discharge, or invasive ventilation). For the conditional outcome model, we desired a modelling approach that could accommodate interactions, nonlinearity, provided posterior distributions to maintain the Bayesian approach, and had a demonstrated record of efficacy in observational causal inference. Bayesian additive regression trees fulfill these criteria.

7.1 Bayesian additive regression trees in causal inference

Bayesian additive regression trees (BART) is a non-parametric tree-based regression technique that uses Bayesian prior distributions to favour small trees with regularized leaf weights. There are many helpful reviews on the method (24,25). BART has several advantages in causal modelling: it can handle a large set of confounders with potential interactions among confounders as well as non-linear relationships between confounders and outcome, and it's a simpler approach that requires less model specification in model fitting .

7.2 Model description and computational details

For this study, we used BART for probit regression.(27,28) We used Dirichlet prior distributions to encourage parsimonious trees (29). The model drew 800 posterior samples of 200 trees each, used a burn-in of 250 samples, and thinned to keep 1 of every 50 samples in order to achieve good convergence diagnostics.(27) The primary outcome model focused on time to death in hospital, and assumed that if patients were discharged from hospital then they survived until the end of the 28-day observation period. The prior distributions we used included a beta of 2 and alpha of 0.95, which are recognized “default” values for these parameters.

7.3 Covariates

Covariates for the conditional outcome model fell into two categories: baseline and time-varying. The baseline covariates included demographics (age, sex, race/ethnicity where available), admission data (time of admission, length of stay prior to ICU admission, location prior to ICU admission, specific ICU of admission, primary service prior to ICU admission), and most recent clinical data prior to achieving target trial eligibility (vital signs, basic procedures, laboratory values). For the MIMIC cohort we also included comorbidities from the ICD coding at discharge (diabetes, sleep apnea, COPD, congestive heart failure).

Time-varying covariates were features constructed from the observed sequence of confounders. We used the basis functions from the confounder model fit as the time-varying covariates, or "time series features." We used these basis functions because together with the Gaussian process hyperparameters one could reconstruct the entire observed confounder sequence, up to independent identically distributed measurement noise. This made the basis functions an excellent choice to summarise irregularly sampled time series of irregular lengths into a finite set of features that did not depend on the sampling frequency of the data. We used the basis functions from a single randomly selected iteration of the confounder model. We did test model performance with other randomly selected iterations (similar performance) and with an ensemble approach (modest improvement in AUROC of less than 0.01 and in AU-PRC of less than 0.01 on ensemble models of 5, 10, 20 iterations, modest improvement in reliability curve). However, the added computational burden was not worth the minimal improvements in model performance.

7.4 Model outputs and diagnostics

The models were fit using 80 cores on the Compute Canada cluster and took on the order of 1-5 minutes each. Inspection of the Geweke statistics, trace plots, and autocorrelation for keeping every iteration or every 10th iteration showed slightly too much autocorrelation and slightly too many of the patients with Geweke statistic

values beyond the 95th percentile.(27,30) For that reason we kept every 50th iteration. The corresponding Geweke plots, trace plots, and autocorrelations are good and available on request.

7.5 Five-fold cross-validation

We used five-fold cross-validation to measure the discrimination, positive predictive value, specificity, and calibration of the BART model. The results showed good performance for the MIMIC model and less impressive performance for the AmsterdamUMCdb model. The performance was slightly worse than other published models fit using similar durations of observed ICU time series data (31), perhaps because no features incorporated measurement frequency. We did not include features incorporating frequency because we used a standardized observation schedule for the Monte Carlo integration and measurement frequency is not a direct consequence of a patient's physiology.

We compared the model for each cohort with time-varying features to the model for each cohort without time-varying features to show the contribution to model performance attributable to the additional information. Models for both cohorts improved after addition of time-varying features. However, the model for the AmsterdamUMCdb cohort improved less.

7.5.1 Figure e5: Calibration of conditional outcome model

MIMIC

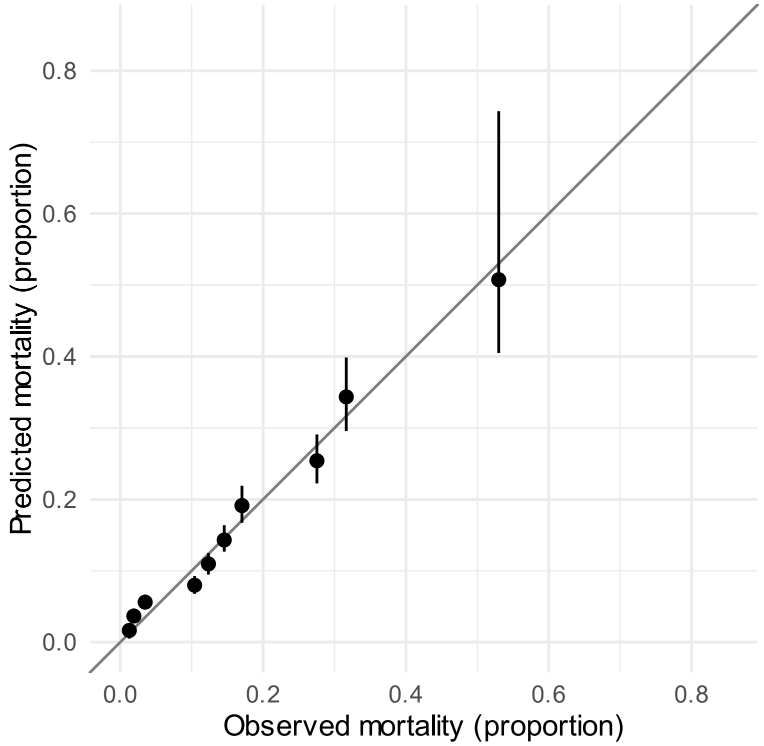

AmsterdamUMCdb

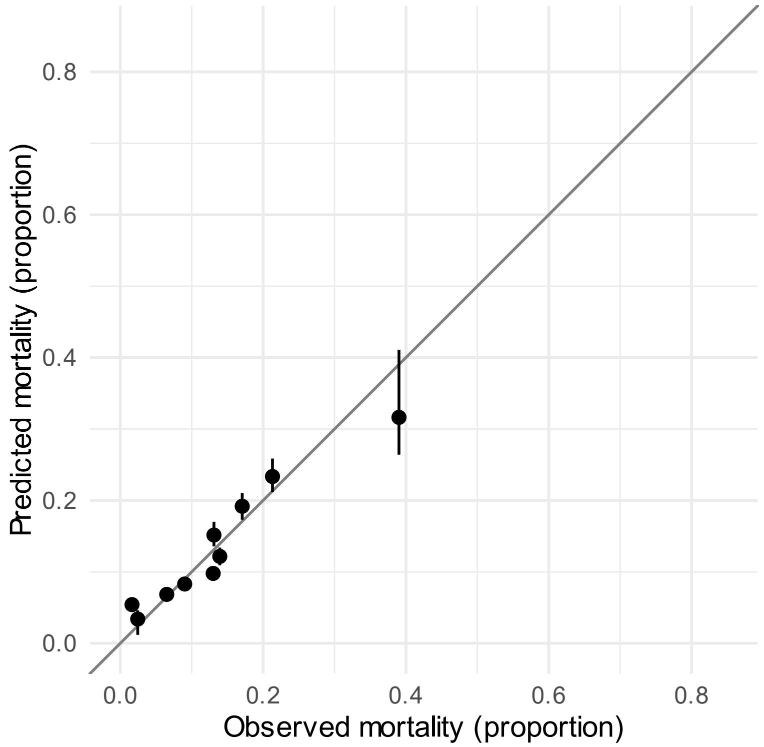

7.5.2 Figure e6: Discrimination and precision of conditional outcome model – MIMIC-IV

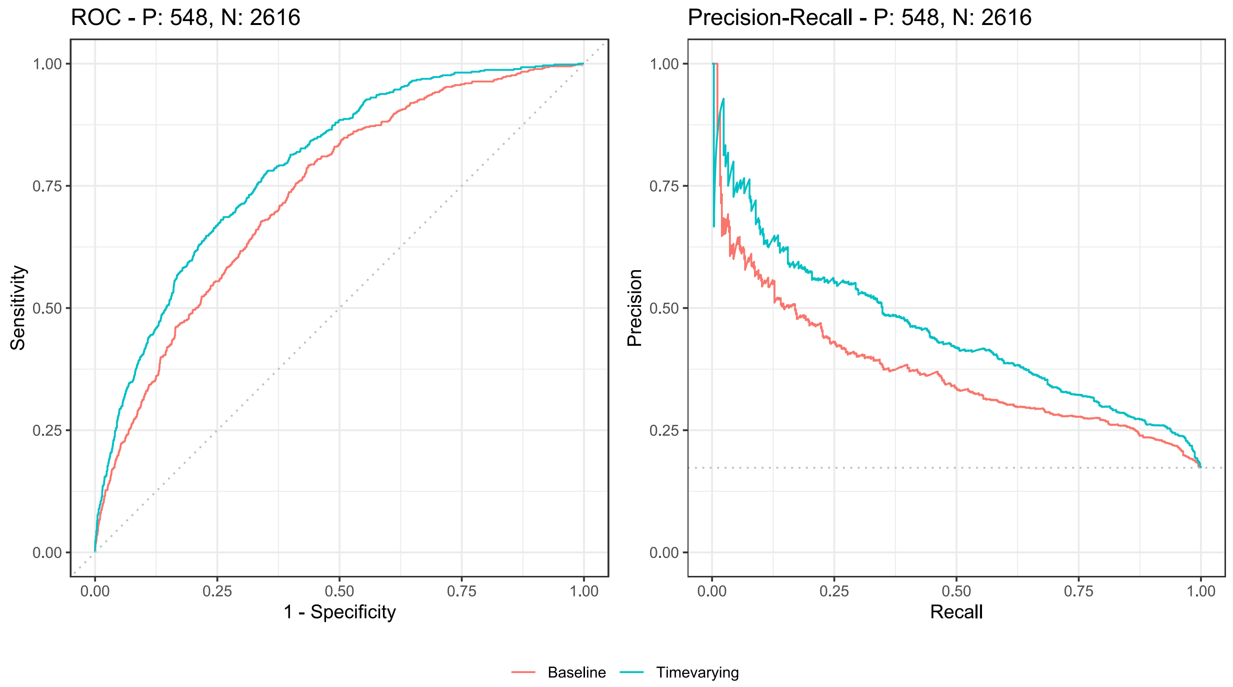

Caption: This figure shows the discrimination (left plot) and precision-recall (positive predictive value versus specificity) for the MIMIC cohort. The blue line denotes the model including time-varying features, while the red line denotes a model which uses only information available at baseline. The discrimination area under the curve for time-varying was 0.789 compared to 0.734 for baseline, while the precisions were 0.45 for time-varying and 0.372 for baseline.

7.5.3 Figure e7: Discrimination and precision of conditional outcome model – AmsterdamUMCdb

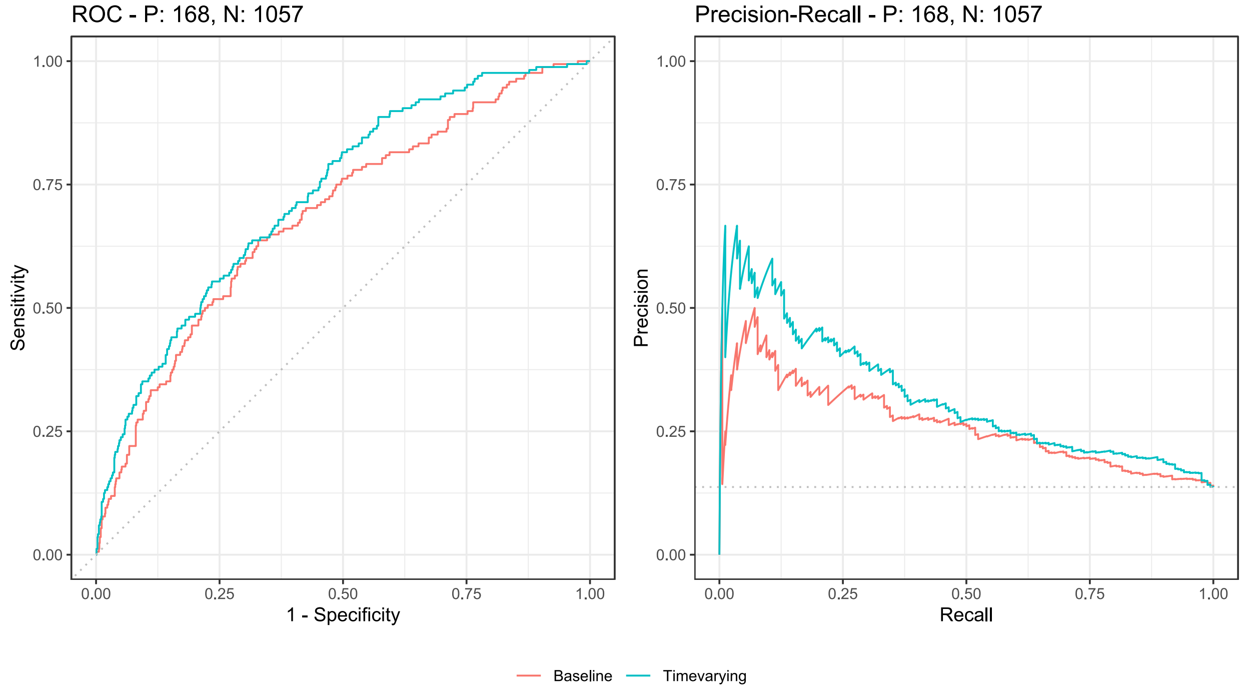

Caption: This figure shows the discrimination and precision-recall for the AmsterdamUMCdb cohort as above. The discrimination area under the curve for time-varying is 0.729 compared to 0.687 for baseline, while the precisions are 0.317 for time-varying and 0.262 for baseline.

8 Monte Carlo integration

The Monte Carlo integration was conducted in a Bayesian fashion as follows:

1) Select a patient
2) Use the confounder model to generate trajectories of time-varying confounders, using that patient's baseline and time-varying variables that were available up to and including the time of eligibility.
3) Apply each threshold to each simulated confounder trajectory
4) Use the conditional outcome model and the simulated confounder trajectory (after applying treatment threshold) to predict the probability of mortality and subsequent invasive ventilation
5) Average over multiple trajectories for the same hyperparameters to obtain one sample of the posterior mean outcomes for that patient
6) Average over all patients to obtain one sample of the posterior mean outcomes for the population
7) Repeat for as many iterations as desired.

8.1 Modeled usual care

To include usual care in our G-computation, we used the binary variable of invasive ventilation from the Gaussian process to model usual care. This variable was the time-varying probability of invasive ventilation according to the observed data ("usual care"). In the usual care threshold, at every measurement, the invasive ventilation variable takes value 0 (no invasive ventilation) or 1 (invasive ventilation). In this way, we could apply usual care to the exact same clinical trajectories on which we tested all of the other thresholds.

8.2 Treatment thresholds and death before invasive ventilation during the target trial period

In the data from both cohorts, some patients died before receiving invasive ventilation. These patients may have been examples of a "failure to rescue" or patients who received palliative care and were either not offered or declined for invasive ventilation. The event of "death before invasive ventilation" was modeled by the Gaussian process. However, No reasonable threshold would allow a patient to die before it was met. Therefore, when the simulated confounder trajectories predicted "death before invasive ventilation" at a given time point, we instead noted the patient as invasively ventilated at that point.

8.3 Simulated trajectories

Below we plot an example simulated confounder trajectory. The confounder model validation plots are also helpful here, because any observed trajectory that can be traced out by the confounder model can also be generated by the confounder model through simulation (it may not be a likely trajectory, but it is possible). The most common discrepancy between simulated trajectories and clinical practice in critical care medicine are the timing of the binary events of ICU discharge or death before invasive ventilation. Both of these events are influenced by non-physiologic variables such as goals of care preferences and ICU / ward bed availability, so it is unsurprising that this was where the simulated trajectories show least realism.

8.4 Figure e8: example simulated trajectory

MIMIC

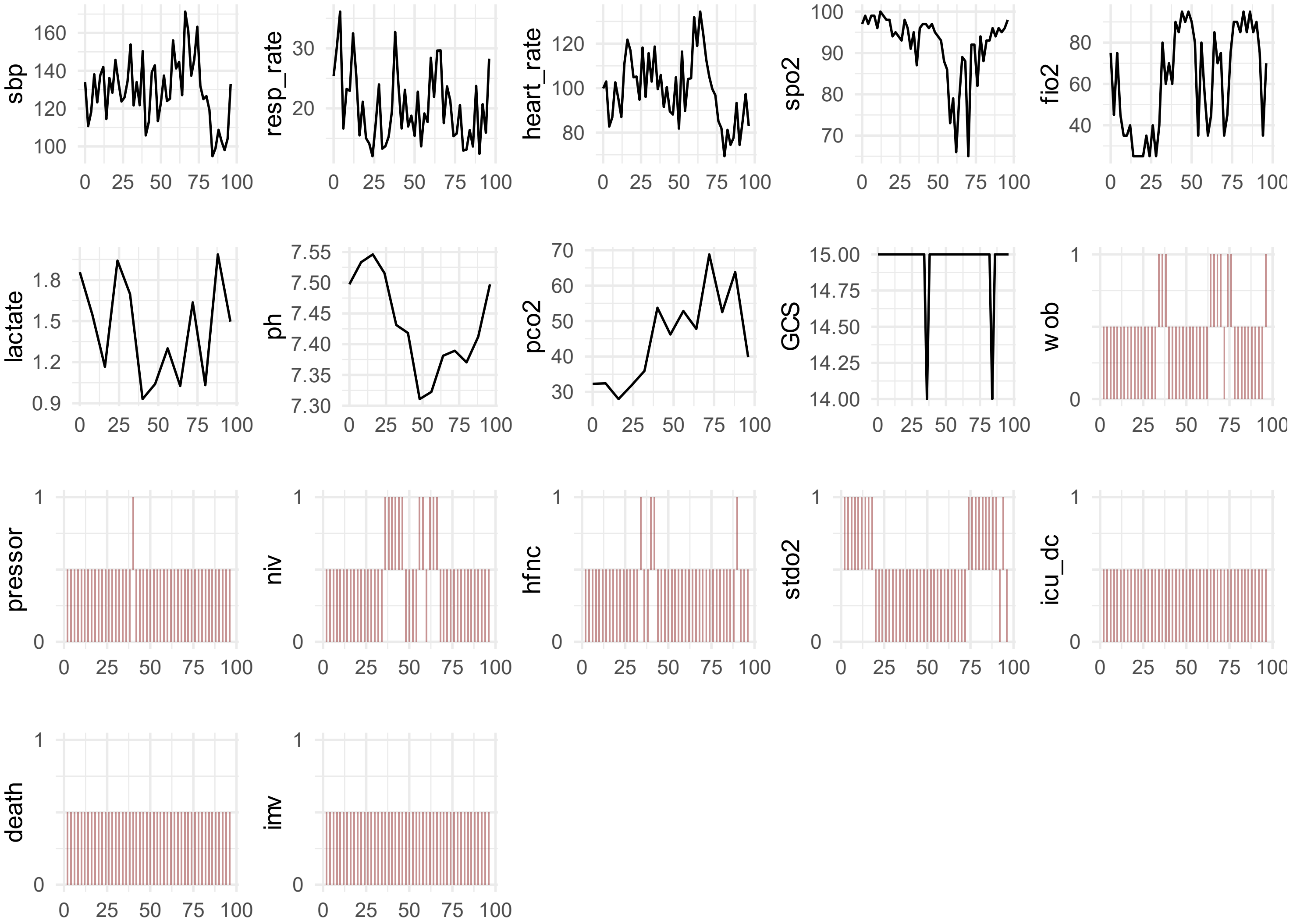

9 Additional results

9.1 Relative risks and e-values

In the primary analysis, compared to threshold SF of less than 88, the relative risk of mortality was 0.94 (CrI 0.90 to 0.98) with threshold SF less than 98, and 0.86 (0.78 to 0.95) with threshold SF less than 110; the respective e-values for mortality were 1.34 (CrI 1.19 to 1.46) for SF less than 98 and 1.60 (CrI 1.30 to 1.87) for SF less than 110, meaning that unmeasured confounding would require an association between use of the specific threshold and mortality of at least that strength in order to negate the findings.

In the secondary analysis, compared to a threshold SF less than 88, the relative risk of mortality was 1.04 (CrI 1.00 to 1.10) for a threshold SF less than 98, and 1.16 (CrI 1.07 to 1.29) for threshold SF less than 110; the respective e-values were 1.24 (CrI 1.05 to 1.42) for SF less than 98 and 1.58 (CrI 1.34 to 1.89) for SF less than 110.

10 Additional tables and figures

10.1 Table e5: Comparison of MIMIC-IV and AmsterdamUMCdb cohorts and analyses

**Characteristic**	**MIMIC-IV**	**AmsterdamUMCdb**
Available data		
Care limitations	Care limitations recorded for < 5% of patients	Care limitations recorded for 30% of patients, noted as 1 (full code; 57%), 2 (some restrictions such as no dialysis, no CPR, no ventilation; 34%), 3 (no escalation / terminal; 9%)
Comorbidities	Available from discharge summaries	No data
Sociodemographic variables	Sex, age, race/ethnicity, marital status, language ability	Sex and age
Work of breathing	Present	Absent
Oxygen devices	High-flow nasal cannula, non-rebreather, and non-invasive ventilation, facemask, nasal prongs	Same except no high-flow nasal cannula used clinically during the database time period
Clinical practice		
ICU beds / total hospital beds	77/673 (11%)	34/1002 (3.4%)
Patients with respiratory failure	Can be admitted to ICU even without immediate plan for invasive ventilation	ICU admission often precipitated by the decision for invasive ventilation
Modeling		
Confounder model	Weakest performance for predicting binary events (ICU discharge, invasive ventilation, death), and blood gas parameters (pH, pCO2)	Weakest performance for predicting binary events (ICU discharge, invasive ventilation, death). GCS and respiratory rate also have higher error than in the MIMIC-IV confounder model
Conditional outcome model	Discrimination AUROC 0.79, precision AUPRC 0.45	Discrimination AUROC 0.73, precision AUPRC 0.32
Causal inference assumption violations	Positivity (some patients probably have goals of care precluding invasive ventilation), unmeasured confounding	Positivity perhaps less of an issue than for MIMIC-IV (filtered out patients with document goals of care status 3), but unmeasured confounding more of a problem relative to MIMIC-IV due to fewer included confounding variables

10.2 Figure e9: Target trial eligibility flow diagrams

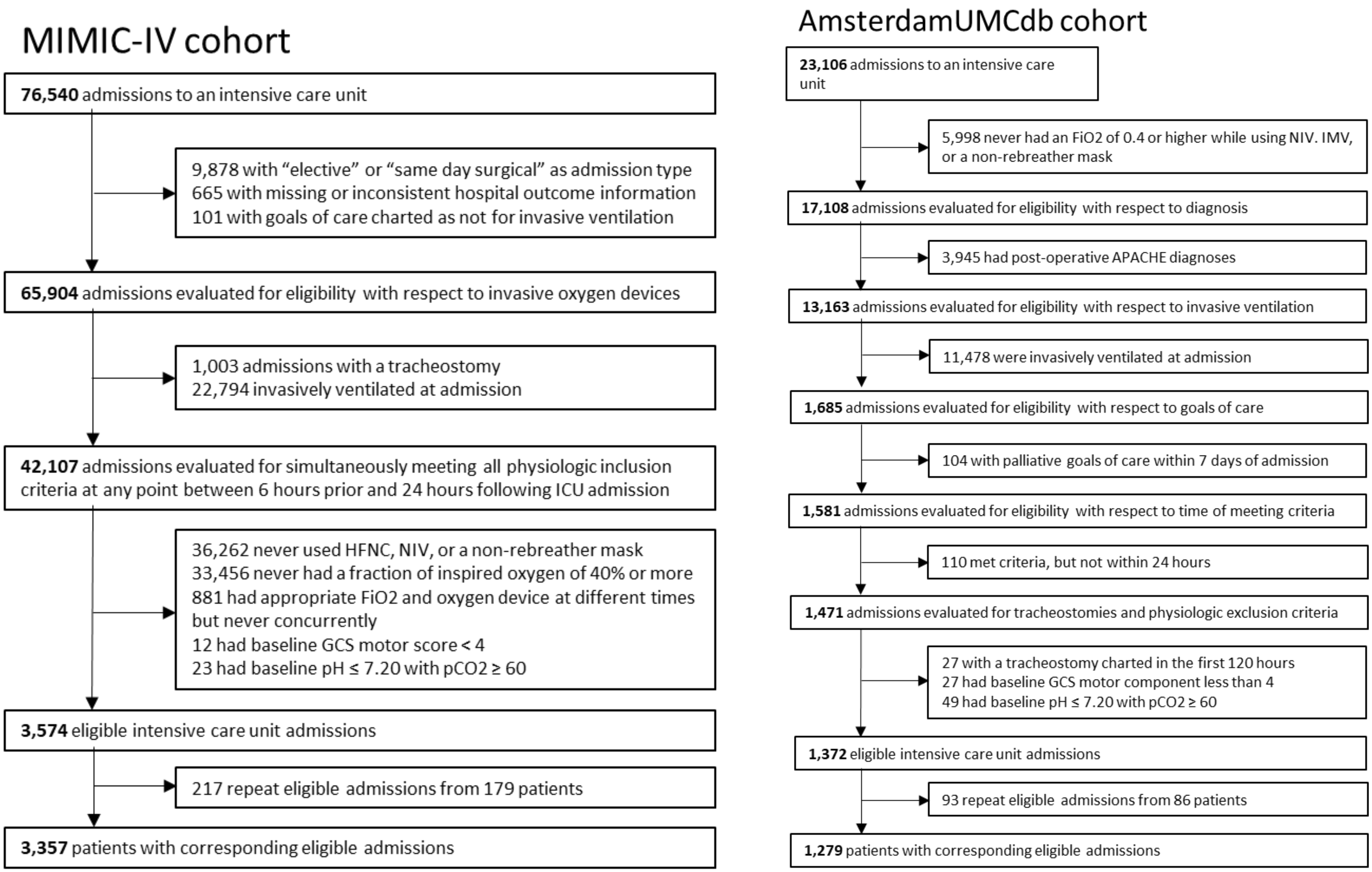

Figure 1 caption: This figure shows the number of patients excluded at each stage of applying the target trial emulation inclusion and exclusion criteria

11 References

1. Darreau C, Martino F, Saint-Martin M, Jacquier S, Hamel JF, Nay MA, et al. Use, timing and factors associated with tracheal intubation in septic shock: a prospective multicentric observational study. Annals of Intensive Care. 2020 Dec;10(1).

2. Roca O, Caralt B, Messika J, Samper M, Sztrymf B, Hernández G, et al. An index combining respiratory rate and oxygenation to predict outcome of nasal high-flow therapy. American Journal of Respiratory and Critical Care Medicine. 2019 Jun;199(11):1368–76.

3. Duan J, Han X, Bai L, Zhou L, Huang S. Assessment of heart rate, acidosis, consciousness, oxygenation, and respiratory rate to predict noninvasive ventilation failure in hypoxemic patients. Intensive Care Med. 2017 Feb 1;43(2):192–9.

4. Frat JP, Thille AW, Mercat A, Girault C, Ragot S, Perbet S, et al. High-flow oxygen through nasal cannula in acute hypoxemic respiratory failure. New England Journal of Medicine. 2015 Jun;372(23):2185–96.

5. Hakim R, Watanabe-Tejada L, Sukhal S, Tulaimat A. Acute respiratory failure in randomized trials of noninvasive respiratory support: A systematic review of definitions, patient characteristics, and criteria for intubation. Journal of Critical Care. 2020 Jun;57:141–7.

6. Grieco DL, Menga LS, Cesarano M, Rosà T, Spadaro S, Bitondo MM, et al. Effect of Helmet Noninvasive Ventilation vs High-Flow Nasal Oxygen on Days Free of Respiratory Support in Patients With COVID-19 and Moderate to Severe Hypoxemic Respiratory Failure: The HENIVOT Randomized Clinical Trial. JAMA. 2021 May 4;325(17):1731–43.

7. de Montmollin E, Aboab J, Ferrer R, Azoulay E, Annane D. Criteria for initiation of invasive ventilation in septic shock: An international survey. Journal of Critical Care. 2016 Feb;31(1):54–7.

8. Bauer PR, Kumbamu A, Wilson ME, Pannu JK, Egginton JS, Kashyap R, et al. Timing of Intubation in Acute Respiratory Failure Associated With Sepsis: A Mixed Methods Study. Mayo Clinic Proceedings. 2017 Oct;92(10):1502–10.

9. Tonelli R, Fantini R, Tabbì L, Castaniere I, Pisani L, Pellegrino MR, et al. Inspiratory Effort Assessment by Esophageal Manometry Early Predicts Noninvasive Ventilation Outcome in de novo Respiratory Failure: A Pilot Study. American Journal of Respiratory and Critical Care Medicine. 2020 Apr;202:558–67.

10. Yarnell CJ, Johnson A, Dam T, Jonkman A, Liu K, Wunsch H, et al. Do thresholds for invasive ventilation in hypoxemic respiratory failure exist? A cohort study. American Journal of Respiratory & Critical Care Medicine. 2022 Sep 14;Accepted.

11. Coudroy R, Frat JP, Girault C, Thille AW. Reliability of methods to estimate the fraction of inspired oxygen in patients with acute respiratory failure breathing through non-rebreather reservoir bag oxygen mask. Thorax. 2020 Sep 1;75(9):805–7.

12. Hernán M, Robins J. Causal Inference: What If. [Internet]. Boca Raton: Chapman & Hall/CRC; 2020. Available from: https://cdn1.sph.harvard.edu/wp-content/uploads/sites/1268/2020/02/ci_hernanrobins_21feb20.pdf

13. Oganisian A, Roy JA. A practical introduction to Bayesian estimation of causal effects: Parametric and nonparametric approaches. Statistics in Medicine. 2021 Jan;40(2):518–51.

14. Delbove A, Darreau C, Hamel JF, Asfar P, Lerolle N. Impact of endotracheal intubation on septic shock outcome: A post hoc analysis of the SEPSISPAM trial. Journal of Critical Care. 2015 Dec;30(6):1174–8.

15. Frat JP, Ragot S, Coudroy R, Constantin JM, Girault C, Prat G, et al. Predictors of intubation in patients with acute hypoxemic respiratory failure treated with a noninvasive oxygenation strategy. Critical Care Medicine. 2018 Feb;46(2):208–15.

16. Bellani G, Laffey JG, Pham T, Madotto F, Fan E, Brochard L, et al. Noninvasive Ventilation of Patients with Acute Respiratory Distress Syndrome: Insights from the LUNG SAFE Study. American Journal of Respiratory and Critical Care Medicine. 2017 Jan;195(1):67–77.

17. Gelman A, Carlin JB, Stern HS, Dunson DB, Vehtari A, Rubin DB. Bayesian Data Analysis, Third Edition. CRC Press; 2013. 677 p.

18. Betancourt M. Robust Gaussian Process Modeling [Internet]. [cited 2021 Oct 9]. Available from: https://betanalpha.github.io/assets/case_studies/gaussian_processes.html#1_Modeling_Functional_Relationships

19. Riutort-Mayol G, Bürkner PC, Andersen MR, Solin A, Vehtari A. Practical Hilbert space approximate Bayesian Gaussian processes for probabilistic programming [Internet]. arXiv; 2022 Mar [cited 2022 May 17]. Report No.: arXiv:2004.11408. Available from: http://arxiv.org/abs/2004.11408

20. Moore L, Lavoie A, Camden S, Le Sage N, Sampalis J, Bergeron E, et al. Statistical Validation of the Glasgow Coma Score. The Journal of trauma. 2006 Jul 1;60:1238–43; discussion 1243.

21. Sackett DL, Gibson RW, Bross IDJ, Pickren JW. Relation between Aortic Atherosclerosis and the Use of Cigarettes and Alcohol. New England Journal of Medicine. 1968 Dec 26;279(26):1413–20.

22. Cheng LF, Dumitrascu B, Darnell G, Chivers C, Draugelis M, Li K, et al. Sparse multi-output Gaussian processes for online medical time series prediction. BMC Medical Informatics and Decision Making. 2020 Jul 8;20(1):152.

23. Carpenter B, Gelman A, Hoffman MD, Lee D, Goodrich B, Betancourt M, et al. Stan: A probabilistic programming language. Journal of Statistical Software. 2017 Jan;76(1):1–32.

24. Chipman HA, George EI, McCulloch RE. BART: Bayesian additive regression trees. Ann Appl Stat. 2010;4(1):266–98.

25. Hill J, Linero A, Murray J. Bayesian Additive Regression Trees: A Review and Look Forward. Annual Review of Statistics and Its Application. 2020 Mar;7(1):251–78.

26. Hahn PR, Dorie V, Murray JS. Atlantic Causal Inference Conference (ACIC) Data Analysis Challenge 2017. arXiv [Internet]. 2019 May; Available from: http://arxiv.org/abs/1905.09515

27. Sparapani RA, Logan BR, McCulloch RE, Laud PW. Nonparametric survival analysis using Bayesian Additive Regression Trees (BART). Stat Med. 2016 Jul 20;35(16):2741–53.

28. Sparapani R, Logan BR, McCulloch RE, Laud PW. Nonparametric competing risks analysis using Bayesian Additive Regression Trees. Stat Methods Med Res. 2020 Jan 1;29(1):57–77.

29. Linero AR. Bayesian Regression Trees for High-Dimensional Prediction and Variable Selection. Journal of the American Statistical Association. 2018 Apr;113(522):626–36.

30. Sparapani R, Dabbouseh NM, Gutterman D, Zhang J, Chen H, Bluemke DA, et al. Detection of Left Ventricular Hypertrophy Using Bayesian Additive Regression Trees: The MESA (Multi-Ethnic Study of Atherosclerosis). Journal of the American Heart Association. 2019 Mar 5;8(5):e009959.

31. Thorsen-Meyer HC, Nielsen AB, Nielsen AP, Kaas-Hansen BS, Toft P, Schierbeck J, et al. Dynamic and explainable machine learning prediction of mortality in patients in the intensive care unit: a retrospective study of high-frequency data in electronic patient records. The Lancet Digital Health. 2020 Apr 1;2(4):e179–91.
